# Whole-genome sequencing of *Fusarium* spp. causing sugarcane root rot on both chewing cane and sugar-making cane

**DOI:** 10.1007/s44154-023-00145-7

**Published:** 2024-01-25

**Authors:** Xinyang Li, Yuming Ma, Na Zhang, Yiming Li, Zhibin Liang, Yibao Luo, Longxin Lin, Dongliang Zhang, Yongqiang He, Ziting Wang, Zhiquan Zhang, Yizhen Deng

**Affiliations:** 1 State Key Laboratory for Conservation and Utilization of Subtropical Agro-Bioresource, Guangxi Key Laboratory of Sugarcane Biology, Guangxi University, Nanning, 530004 China; 2 State Key Laboratory for Conservation and Utilization of Subtropical Agro-Bioresources, Guangdong Province Key Laboratory of Microbial Signals and Disease Control, South China Agricultural University, Guangzhou, 510642 China

**Keywords:** Sugarcane root rot, *Fusarium*, Different inoculation methods, Pathogenicity, Whole-genome sequencing, Evolutionary analysis

## Abstract

Previously we isolated three *Fusarium* strains (a *F. sacchari* strain namely GXUF-1, and another two *F. commune* strains namely GXUF-2 and GXUF-3), and we verified that GXUF-3 was able to cause sugarcane root rot to the chewing cane cultivar Badila. Considering that *Fusarium* spp. are a group of widely distributed fungal pathogens, we tested whether these three *Fusarium* isolates were able to cause root rot to Badila as well as sugar-making cane cultivar (Guitang42), using a suitable inoculation method established based on infection assays using Badila. We found that the three *Fusarium* strains were able to cause root rot symptoms to both Badila and Guitang42, to different extents. To better investigate the potential pathogenicity mechanisms, we performed Illumina high-throughput sequencing and analyzed the whole genomic sequence data of these three *Fusarium* strains. The results reveal that the assembly sizes of the three *Fusarium* strains were in a range of 44.7–48.2 Mb, with G + C contents of 48.0–48.5%, and 14,154–15,175 coding genes. The coding genes were annotated by multiple public databases, and potential pathogenic genes were predicted using proprietary databases (such as PHI, DFVF, CAZy, etc.). Furthermore, based on evolutionary analysis of the coding sequence, we found that contraction and expansion of gene families occurred in the three *Fusarium* strains. Overall, our results suggest a potential risk that the root rot disease may occur to the sugar-making canes although it was initially spotted from fruit cane, and provide clues to understand the pathogenic mechanisms of *Fusarium* spp. causing sugarcane root rot.

**Supplementary Information:**

The online version contains supplementary material available at 10.1007/s44154-023-00145-7.

## Introduction

Sugarcane is an important crop widely grown in tropical and subtropical regions, serving as raw materials for sugar and energy industry (Manners and Casu 2011). In recent years, the long-term ratoon planting mode of sugarcane has resulted in accumulation of pathogen(s) in the soil, thus causing a significant decline in the yield and quality of sugarcane (Pang et al. 2021; Ren et al. 2021). Sugarcane root rot is an emerging and serious soil-borne root disease, which could occur throughout the entire growth period of sugarcane. At the early stage of the disease, the aboveground parts of sugarcane plants are short, and the underground root system gradually turns brown, soft, and rotten. Later, the ability of the root system to absorb water and nutrients decreases, resulting in leaves curling, yellowing, and even wilting. The whole sugarcane plant would die in severe cases (Ren et al. 2021). Field surveys have found that the onset of sugarcane root rot can reduce yields by an average of 30% to 50% (Ren et al. 2022). Therefore, the occurrence of root rot is one of the main causes for the sugarcane succession disorder.

*Fusarium commune* was characterized as the causal pathogen of sugarcane root rot mainly based on its morphological characteristics, molecular identification using rDNA internal transcribed spacer (rDNA-ITS) and elongation factor 1-alpha (EF-1alpha), and its infection in fruit cane cultivar Badila (Wang et al. 2018; Li et al. 2022). In our previous study, besides *F. commune* GXUF-3, another two *Fusarium* strains, namely GXUF-1 and GXUF-2, were also isolated from the rot root of sugarcane. It awaits to confirm whether they are capable of causing the root rot disease. Although the root rot disease and the causal pathogen was initially reported in fruit cane (cultivar Badila) (Wang et al. 2018), it cannot rule out that the disease may also occur to the sugar-making canes. Therefore, to test whether these three isolated *Fusarium* strains are also pathogenic to other sugarcane cultivars (e.g., sugar-making cane Guitang42 or ROC 22), we need to establish an appropriate indoor inoculation method. The reported indoor inoculation methods used for testing *Fusarium* spp. pathogenicity include direct watering fungal liquid (DWFL), fungal liquid mixed with soil (FLMWS), direct inoculation fungal cultures (DIFC), fungal cultures mixed with soil (FCMWS), root soaking (RS), injection, and spraying (Ren et al. 2021; Zhou et al. 2022). DWFL method is commonly used for testing the pathogenicity of *Fusarium* spp. on various crops such as sugarcane root rot (Ren et al. 2021), watermelon *Fusarium* wilt (Zhou et al. 2022), and cucumber *Fusarium* wilt (Zhou et al. 2010).

Genomic analysis provides clues for identification of potential pathogenic factors, and thus for understanding pathogenic mechanisms of plant pathogens. Genomic sequencing and analysis have been performed in multiple pathogenic *Fusarium* species, including *F. oxysporum* causing root rot in *Panax notoginseng* (Wen et al. 2020), *F. solani-melongenae* causing root and stem rot in sweetpotato (Xie et al. 2022), and *F. commune* causing lotus rhizome rot (Kuang et al. 2022). A lot more other *Fusarium* spp. causing diseases to the crop plants have not been sequenced and analyzed on the whole-genome level.

In this study, we tested pathogenicity of the three isolated *Fusarium* strains on the sugar-making cane cultivar Guitang42, using an optimal inoculation method evaluated on Badila. All the three *Fusarium* strains were able to cause root rot symptoms to Guitang42, indicating a potential risk of this disease spreading to sugar-making sugarcane. To further investigate species classification, potential pathogenic mechanisms, and evolution of gene families, on the genomic level, we sequenced the genomes of these three strains, and performed functional annotation, prediction of pathogenicity factors, and evolutionary analyses (Experimental design was illustrated as in Fig. S1). Overall, our study not only provides insights into the function and regulatory mechanism of pathogenic genes in *Fusarium* spp. potentially contributing to disease occurrence and/or progression, but also provides reference for selection and breeding of new disease-resistant varieties of sugarcane.

## Results

### Three *Fusarium* strains caused sugarcane root rot to the chewing cane Badila

**Fig. 1:**
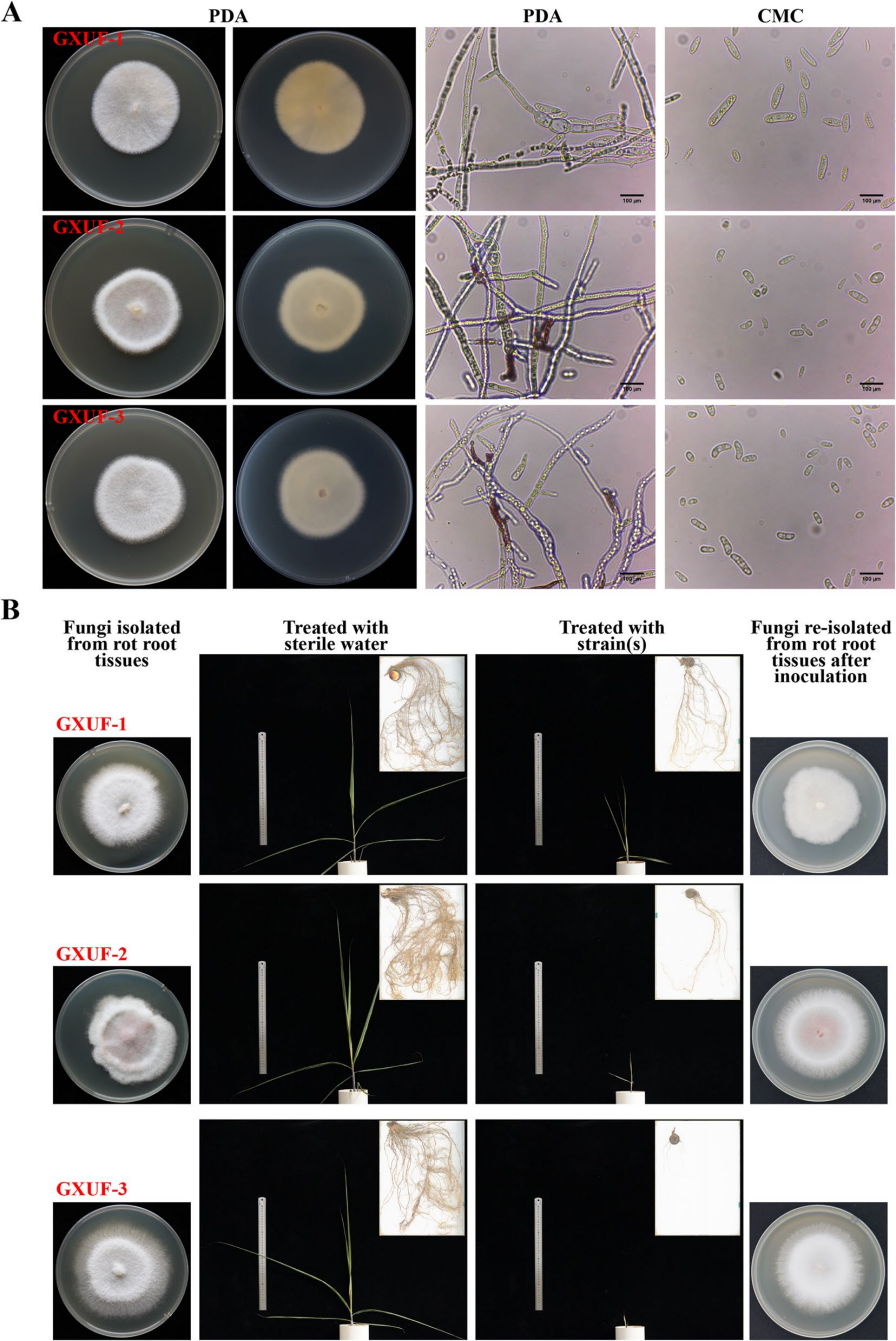
Isolation and identification of sugarcane root rot pathogen(s). **A** Front and back morphology colony grown on PDA medium and microscopic observation of mycelia and spores for GXUF-1, GXUF-2, and GXUF-3 cultured on PDA medium (mycelia) and in CMC medium (spores). Scale bar = 100 μm. **B** Koch’s postulates validation of three strains (GXUF-1, GXUF-2, and GXUF-3) as the causal pathogens of sugarcane root rot

Three fungal strains were isolated and purified from the diseased root samples, namely GXUF-1, GXUF-2, and the reported GXUF-3 (Li et al. 2022). In spite of the diversity in colony morphology, pigment production, and sporulation type, these three strains displayed typical characteristics of the *Fusarium* genus (Fig. 1A). Colony morphology analysis showed that after grown on Potato Dextrose Agar (PDA) medium for 5 d, the hyphae of these three fungal strains were fluffy and grew close to the medium surface. GXUF-1 and GXUF-3 formed white velvet-like mycelial colony, whereas the GXUF-2 had micro-pink velvet-like mycelial colony (Fig. 1A). All these strains appeared white or pale-yellow when viewed from the backside (Fig. 1A). Some of GXUF-2 or GXUF-3 hyphae produced red pigment, which were not observed in the hyphae of GXUF-1 (Fig. 1A). Sporulation type analysis showed that these fungal strains produced a large number of small conidia, but less large conidia when cultured on PDA medium. However, abundant large conidia of sickle- or spindle-shape with 0 to multiple septa per conidium, and small conidia of oval with 0–1 septa per conidium, were produced when cultured in liquid carboxymethyl cellulose (CMC) medium (Fig. 1A).

To verify whether these three strains are the causal pathogens of sugarcane root rot, we inoculated them individually in the sterilized soils that were used for planting sugarcane (Badila). After 45 days, morphology of the aboveground plantlets and underground root system of sugarcane was photography, and the underground part of the inoculated sugarcane plantlets were dug out for re-isolation of fungal pathogens. We observed that the aboveground part of the sugarcane plantlets inoculated with these three *Fusarium* strains was dwarfed, and the leaves turned yellow, and meanwhile the infect sugarcane roots were seriously hindered, soft, and rotten (Fig. 1B). In contrast, the sugarcane inoculated with sterile water (untreated control) grew normally and showed no disease symptoms (Fig. 1B). The fungal stains showing similar colony morphology could be isolated from the underground part of inoculated and diseased plants (Fig. 1B). The above findings confirmed that GXUF-1, GXUF-2, and GXUF-3 were the causal pathogens of sugarcane root rot.

### Evaluation of indoor inoculation methods using Badila

To establish a suitable indoor inoculation method for assessing pathogenicity of these three strains, we quantitatively evaluated total root length, total root volume, and total root surface area (detailed procedure described in Materials and Methods), which were commonly used as indicators to evaluate the root absorptive function (Deng et al. 2020; Li et al. 2022). GXUF-1, GXUF-2, and GXUF-3 were inoculated into healthy sugarcane tubers according to different methods (DWFL, FLMWS, DIFC, and FCMWS, respectively; Fig. S1B). The results showed that inoculated sugarcane seedlings displayed the aboveground phenotype (reflected by changes in plant height, fresh weight, and dry weight) and root phenotype (reflected by changes in total root length, total root surface area, and total root volume), of different extents under different inoculated methods. The disease phenotypes were significantly affected by different strains and inoculation methods (Tables S1 and S2). Two-factor (strain and inoculation method) analysis showed that inoculation of GXUF-3 with DWFL method caused the most severe aboveground phenotypes and root phenotypes, as no seedling or root growth at all (Tables S1 and S2).

Two-factor (strain and inoculation method) analysis of the physicochemical property of the rhizosphere soil showed that the content of macro-elements (TC, total carbon; TN, total nitrogen; AP, available phosphorus; AK, available potassium) almost all increased in the *Fusarium* strain inoculated rhizosphere soil, especially for TC content (Table S3). Consistently, inoculation of GXUF-3 using DWFL method caused the greatest changes in TC, TN, AP, and AK (Table S3). However, different *Fusarium* strains caused no significant difference in AP or AK change in rhizosphere soil under each inoculation method. Also, there was no significant interaction between the two factors, strain and inoculation method, in TC, TN, AP, and AK contents in rhizosphere soil. Micro-elements (Ca, calcium; Mg, Magnesium; Mn, Manganese; Fe, Iron; Cu, Copper) in sugarcane rhizosphere soil, were also affected by strains and inoculation methods (Table S3).

Overall, based on the above analyses, we chose DWFL as a suitable method for indoor inoculation to assess the pathogenicity of *Fusarium* strains to sugar-making cultivar.

### Pathogenicity assay of *Fusarium* strains on sugar-making cultivar Guitang42 using DWFL method

**Fig. 2:**
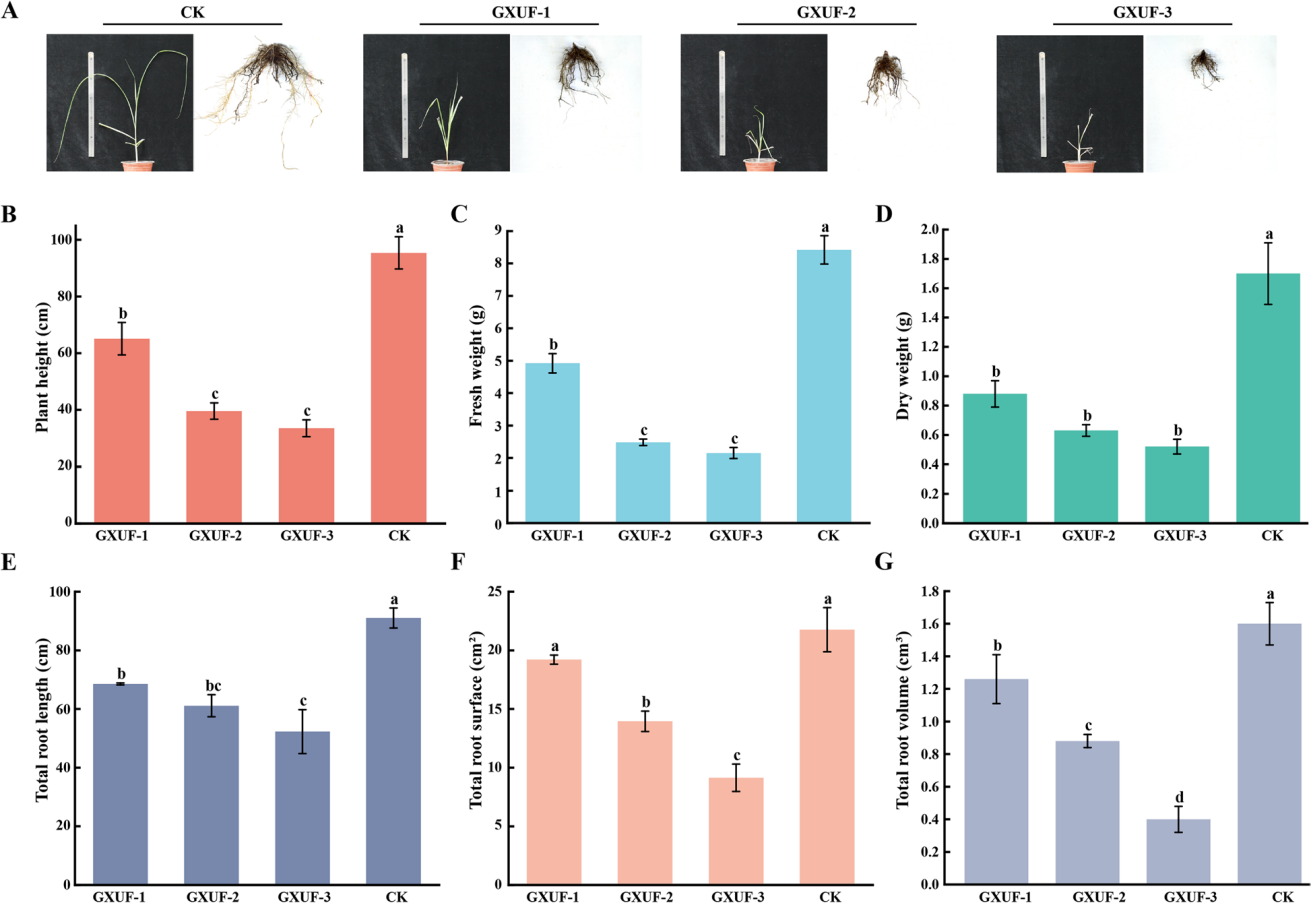
Pathogenicity assay of *Fusarium* strains on sugar-making cultivar Guitang42 using DWFL method. **A** The aboveground plantlets and underground root phenotypes of sugarcane (making sugar) inoculated with the three *Fusarium* pathogens causing root rot using the DWFL method after 45 days post-inoculation. CK represented non-inoculated. Each treatment was repeated 3 times (*n* = 3). The aboveground phenotypes of Guitang42, including plant height (**B**), fresh weight (**C**), and dry weight (**D**), were assessed for the Guitang42 inoculated with *Fusarium* strain or without (CK), respectively. The underground phenotypes of Guitang42, including total root length (**E**), total root surface (**F**), and total root volume (**G**)**,** were assessed for the Guitang42 inoculated with *Fusarium* strain or without (CK), respectively. Statistical analyses in (**B**-**G**) were performed by SPSS V27 software based on analysis of variance (ANOVA) followed by Duncan’s test

We inoculated the three strains to sugar-making cane Guitang42 using DWFL method. After grown for 45 days, the aboveground of the infected sugarcane displayed dwarfed seedlings and yellowish leaves (Fig. 2A), as well as decreased in plant height, fresh weight, and dry weight (Fig. 2B-D). Meanwhile, the infect sugarcane roots were short and soft (Fig. 2A). Quantification analysis showed that total root length, total root surface area, and total root volume were all significantly lower in the infected plants than those in the untreated sugarcane (Fig. 2E-G). Contrary to the infected sugarcane, the untreated plants (control) grew normally and showed no disease symptoms in either aboveground or underground parts (Fig. 2A-G). GXUF-3 caused the strongest symptoms among the three tested strains. Overall, we confirmed that GXUF-1, GXUF-2, and GXUF-3 are capable of causing sugarcane root rot on chewing cane Badila as well as sugar-making sugarcane Guitang42.

### Assembly and component analysis of genome sequences

As the three *Fusarium* strains pose a potential risk to the sugarcane production and sugar industry, it is necessary to investigate the pathogenicity mechanisms of them. The genomes of GXUF-1, GXUF-2, and GXUF-3 were sequenced using Illumina sequencing technology, and the raw data were subjected to quality control using Fastp software (https://github.com/OpenGene/fastp) to clear out the sequencing adapter and low-quality reads (Fig. S1C). The quality of all samples before and after filtration was summarized in Table S4. GXUF-1, GXUF-2, and GXUF-3 were assembled into 2095, 6890, and 4726 contigs (N50 sizes of 73.1, 18.6, and 93.7 kb), and the draft genome sizes were 44.7, 47.3, and 48.2 Mb, with G + C contents of 48.5, 48.0, and 48.0%, respectively (Table 1). The Benchmarking Universal Single-Copy Orthologs (BUSCO) genome completeness evaluation illustrated that 98.0%, 96.1%, and 97.7% of the genome integrity were obtained, respectively (Table 1). Average Nucleotide Identity (ANI) analysis showed that the nucleotide sequence of GXUF-1 had the highest similarity (96.89%) to that of *F. sacchari* FS66, while the remaining two strains had the highest nucleotide sequence similarity to *F. commune* F23a (99.23% and 99.19%, respectively) (Table 1) (Fig. S2A).

**Table 1:** Genome assembly statistics

Features	GXUF-1	GXUF-2	GXUF-3
Assembly size (bp)	44,739,610	47,303,747	48,236,101
Contigs	2095	6890	4726
Contigs N50 (kb)	73.1	18.6	93.7
G + C content (%)	48.5	48.0	48.0
BUSCO assessment (%)	98.0	96.1	97.7
Average Nucleotide Identity (ANI)	*F. sacchari* FS66 (96.89)	*F. commune* F23a (99.23)	*F. commune* F23a (99.19)

Alignment of repetitive sequence type between two different genomes can reflect the rate of divergence between two different species (Zhang et al. 2022). The total lengths of repeated sequences predicted in GXUF-1, GXUF-2, and GXUF-3 genomes were 872,479, 1,648,411, and 1,861,211 bp, accounting for 1.95, 3.48, and 3.86% of the whole-genome sequences, respectively (Table 2). In the prediction of repetitive sequences, long terminal repeats (LTRs), long/short interspersed nuclear elements (LINEs/SINEs), rolling circles (RCs), satellites, and simple repeats accounted for less than 1% of each of the examined genome. Among them, total numbers of 312, 729, and 1132 LTRs with total lengths of 97,350, 176,277, and 254,937 bp respectively found in GXUF-1, GXUF-2, and GXUF-3, accounted for less than 0.60% of each genome. LINEs were not predicted in GXUF-1, while 471 and 343 LINEs were found in GXUF-2 and GXUF-3, respectively, with total lengths of 166,765 and 118,618 bp, accounting for 0.35% and 0.25% of their genomes, respectively (Table 2). Furthermore, SINEs, RCs, and satellites were less than 0.1% of the genomes of the three strains, namely SINEs (0.01, 0.02, and 0.01%), RCs (0.00, 0.09, and 0.07%) and satellites (both were 0.02%). GXUF-2 and GXUF-3 contain a total number of 1950 and 1905 DNA transposons, with a total length of 523,878 and 569,125 bp, both exceeding 1% of the genome, but no DNA transposons were predicted in GXUF-1 (Table 2). The number of protein-coding genes was 14,154, 15,175, and 14,820 in GXUF-1, GXUF-2, and GXUF-3, respectively. The total lengths of protein-coding genes were 23,803,595, 23,888,055, and 24,281,677 bp, with the average length as 1681.76, 1574.17, and 1638.44 bp, respectively (Table 2). The total lengths of the coding regions of the three tested strains accounted for more than 50% of the genome (Table 2). Distribution of gene lengths was shown in Fig. S2B, among which the number of genes larger than 2500 bp was the largest. The number of the genes larger than 2500 bp in GXUF-1, GXUF-2, and GXUF-3 was 2461, 2283, and 2446, respectively.

**Table 2:** Genome components analysis

Prediction features			GXUF-1	GXUF-2	GXUF-3
Repeat sequence	Total repetitive sequence length (bp)		872,479	1,648,411	1,861,211
	Repetitive sequence content (%)		1.95	3.48	3.86
	Long terminal repeats (LTRs)	Number	312	729	1132
		Total length (bp)	97,350	176,277	254,937
		In genome (%)	0.22	0.37	0.53
	DNA transposons	Number	–	1950	1905
		Total length (bp)	–	523,878	569,125
		In genome (%)	–	1.11	1.18
	Long interspersed nuclear elements (LINEs)	Number	–	471	343
		Total length (bp)	–	166,765	118,618
		In genome (%)	–	0.35	0.25
	Short interspersed nuclear elements (SINEs)	Number	84	107	52
		Total length (bp)	6520	8147	3828
		In genome (%)	0.01	0.02	0.01
	Rolling circles (RCs)	Number	19	462	346
		Total length (bp)	1367	41,374	32,866
		In genome (%)	0.00	0.09	0.07
	Satellites	Number	89	103	103
		Total length (bp)	8304	9098	9421
		In genome (%)	0.02	0.02	0.02
	Simple repeats	Number	8209	6149	6356
		Total length (bp)	318,156	247,790	255,698
		In genome (%)	0.71	0.52	0.53
	Unknown	Number	3457	3604	4113
		Total length (bp)	391,128	429,266	575,240
		In genome (%)	0.87	0.91	1.19
Coding gene	Protein-coding genes number		14,154	15,175	14,820
	Total gene length (bp)		23,803,595	23,888,055	24,281,677
	Average gene length (bp)		1681.76	1574.17	1638.44
	Gene length / Genome (%)		53.20%	50.50%	50.34%
	Exon number		40,918	42,458	42,709
	Start codon number		13,986	14,252	14,537
	Stop codon number		13,987	14,216	14,529
Non-coding RNA	ribozyme		2	3	3
	antisense		1	–	1
	tRNA		283	272	302
	rRNA		96	82	96
	sRNA		3	4	4
	snRNA		37	31	36
	miRNA		–	–	1
	others		8	3	12
Pseudogene	Pseudogene number		13,912	12,944	14,859
	Total pseudogene length (bp)		21,063,084	18,390,751	21,594,373
	Average pseudogene length (bp)		1514.02	1420.79	1453.29

“---” stands for non-existence

The ncRNAs mainly include transfer RNAs (tRNAs), ribosomal RNAs (rRNAs), small RNAs (sRNAs), small nucleolar RNAs (snRNAs), and microRNAs (miRNAs) (Li et al. 2013). GXUF-1, GXUF-2, and GXUF-3 contain predicted tRNAs (283, 272, and 302), rRNAs (96, 82, and 96), sRNAs (3, 4, and 4), snRNAs (37, 31, and 36). Notably, only one miRNA was predicted in GXUF-3 (Table 2). Pseudogenes are genes that have similar sequences to protein-encoding genes but have lost their original functions due to mutations such as insertions and deletions (Ma et al. 2021). The number of pseudogenes predicted for GXUF-1, GXUF-2, and GXUF-3 was 13,912, 12,944, and 14,859, respectively, with a total length of 21,063,084, 18,390,751, and 21,594,374 bp, respectively, and an average length longer than 1420 bp (Table 2).

### Gene functional annotation by general databases

Using the Eukaryotic Orthologous Groups (KOG) database, 10,664, 11,686, and 11,402 genes were annotated for GXUF-1, GXUF-2, and GXUF-3 (Fig. S2C), respectively, exceeding 75% of the predicted gene numbers of each genome. The protein functions of the three strains were mainly focused on carbohydrate transport and metabolism, secondary metabolites biosynthesis, transport and catabolism, post-translational modification, protein turnover, chaperones, and amino acid transport and metabolism, as summarized in Fig. S3.

In Gene Ontology (GO) database, 3971, 4275, and 4075 genes were annotated respectively for GXUF-1, GXUF-2, and GXUF-3 (Fig. S2C), exceeding 27% of the predicted gene numbers of each genome. The encoded protein sequences could be classified into three major GO categories, Biological Process (BP), Molecular Function (MF), and Cellular Component (CC). Number of sub-categories were displayed in Table S5. Cellular process contains the most coding genes in the BP category for these three strains, respectively as 3599, 3884, and 3696 genes. In MF category, catalytic activity contains the most coding genes as 1884, 2024, and 1934, respectively. In CC category, “cell” and “cell parts” terms contain the most coding genes as 3592, 3871, and 3682 genes, respectively (Table S5). Additionally, the respective major GO categories of GXUF-1, GXUF-2, and GXUF-3 were analyzed for shared genes, which were found to be 2996, 3239, and 3082, respectively (Fig. S4A). Subsequently, the protein-encoding genes of the respective strains were used as background genes and the shared genes as target genes, for GO enrichment analyses. We found that a relatively higher enrichment degree in the BP category than MF or CC category, among these three strains (Fig. S4B).

In Kyoto Encyclopedia of Genes and Genomes (KEGG) database, 4805, 5177, and, 5003 genes were annotated for GXUF-1, GXUF-2, and GXUF-3, respectively (Fig. S2C), exceeding 33% of the predicted gene numbers. The encoded protein sequences of these three genomes were classified into six primary, and 45 secondary taxonomic pathways (under each primary classification) (Fig. S5A-C). Among the primary classifications of Cellular Processes (CP), Environmental Information Processing (EIP), Genetic Information Processing (GIP), Human Diseases (HD), Metabolism (M), and Organismal Systems (OS), M had the highest number of genes (Fig. S5A-C). In primary classifications of M, carbohydrate metabolism contains the most genes, as 527, 600, and 592 in GXUF-1, GXUF-2, and GXUF-3, respectively (Fig. S5A-C; Table S6). In the KEGG global metabolic network, tryptophan metabolism (00380) was found as one of the most disturbed metabolic pathways (Wang et al. 2022), therefore we paid more attention on this pathway in these three genomes. As summarized in Fig. S6, abundant genes were predicted in tryptophan metabolism pathway, indicating that these three *Fusarium* strains were able to produce numerous tryptophan derivative metabolites. We also noticed that conserved genes were predicted in the MAPK signaling pathway (04010), one of the most studied signaling pathways in plant pathogens playing a crucial role in growth development, reproduction, pathogenicity, etc. (Cheng et al. 2012; Peng 2014) (Fig. S7). Additionally, no shared genes were detected among the primary classifications in each individual genome (Fig. S5D).

In Non-Redundant protein sequence (NR) database, 13,209, 15,101, and 13,867 genes were annotated for GXUF-1, GXUF-2, and GXUF-3, respectively (Fig. S2C), exceeding 93% of the predicted number of genes in the whole genomes. According to the number of annotated genes matching the known *Fusarium* species, the top three species for GXUF-1 were *F. proliferatum* (23.89%), *F. mangiferae* (16.20%), and *F. fujikuroi* (15.32%) (Fig. S8A). Similarly, the top three species for both GXUF-2 and GXUF-3 were *F. oxysporum* f. sp. *lycopersici* 4287, *F. oxysporum*, and *F. oxysporum* f. sp. *cubense*, with a slight difference in the number of genes in these two genomes (Fig. S8A). These results indicated that all three strains had a high degree of species homology and a similar evolutionary process with other *Fusarium* species. Overall, the numbers of annotated genes GXUF-1, GXUF-2, and GXUF-3 by the KOG, GO, NR, and KEGG databases were summarized in Fig. S2C, and we noticed that most genes could be annotated in at least two databases.

We next used Protein families database of alignments and hidden Markov models (Pfam) database (Mistry et al. 2021) for predicting protein families and domains. 10,488, 11,468, and 11,205 genes were annotated for GXUF-1, GXUF-2, and GXUF-3, respectively, accounting for 74.10, 75.57, and 75.61% of the predicted genes for each strain, exceeding 74% of the genomes (Fig. S8B). Using the Swiss-Prot database, we annotated 7666, 8082, and 7928 genes for GXUF-1, GXUF-2, and GXUF-3, respectively, accounting for 54.16, 53.26, and 53.50% of the predicted genes for each strain, exceeding 53% of the genomes (Fig. S8B). Cytochrome P-450 (P-450) not only affects secondary metabolites synthesis and metabolism of foreign compounds in fungi, but also mediates biotransformation of variety compounds, thus playing an important role in pesticide degradation (Jiang and Li 2018). Using the P-450 database, we annotated 547, 606, and 584 genes for GXUF-1, GXUF-2 and GXUF-3, respectively, accounting for 3.86, 3.99, and 3.94% of the predicted genes for each strain, less than 4% of the genomes (Fig. S8B).

### Gene functional annotation by proprietary databases

**Fig. 3:**
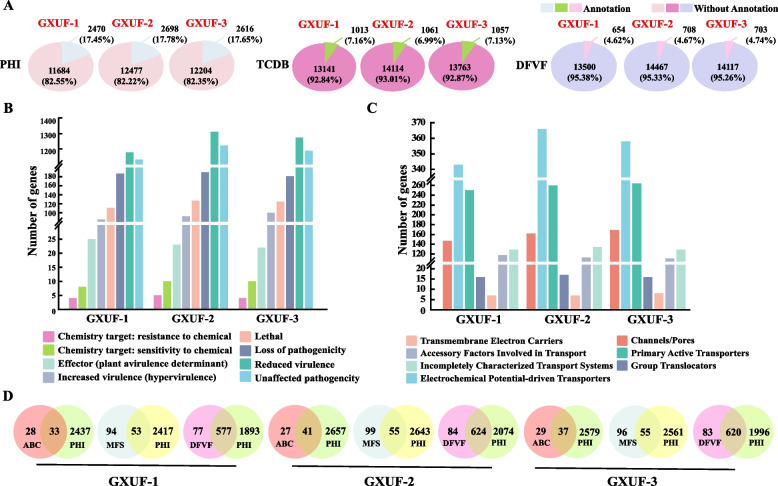
Analysis of the genes annotated by Pathogen-Host Interactions (PHI) database, Transporter Classification Database (TCDB), or Fungal Virulence Factor (DFVF) database in GXUF-1, GXUF-2, and GXUF-3. **A** The pie chart representing the number of annotated genes of these three pathogens causing sugarcane root rot in PHI, TCDB, DFVF databases. **B** PHI annotation statistics of these three pathogens causing sugarcane root rot. **C** TCDB annotation statistics of these three pathogens causing sugarcane root rot. **D** Venn diagrams showing the overlapping of MFS (Major Facilitator Superfamily), ABC (ATP-binding cassette), or DFVF genes with genes annotated in the PHI database in these three pathogens

To investigate the potential pathogenic mechanisms of these three *Fusarium* strains from a genomic view, we used the Pathogen-Host Interactions (PHI) database for gene annotation. A total of 2470, 2698, and 2616 genes was annotated for GXUF-1, GXUF-2, and GXUF-3, respectively, all exceeding 17% of the predicted gene numbers of the respective strain (Fig. 3A). Most of the annotated genes were mainly concentrated in three types, as loss of pathogenicity, reduced virulence, and unaffected pathogenicity (Fig. 3B). These three strains also contain genes annotated as the types of increased pathogenicity (hypervirulence) and effector (plant avirulence determinant), which are relevant to microbial pathogenicity (Fig. 3B). Genes belonging to these two types and with exceeding 90% identity in the three tested strains were listed in Table S7.

Transporter Classification Database (TCDB) is used for predicting genes encoding transporters, which may play a role in microbe-plant interaction (Ai et al. 2021). A total of 1013, 1061, and 1057 genes was annotated for GXUF-1, GXUF-2, and GXUF-3, respectively, accounting for about 7% of the predicted gene numbers of the respective strain (Fig. 3A). Primary classifications of TCDB include Transmembrane Electron Carriers (TECs), Group Translocators (GTs), Accessory Factors Involved in Transport (AFIT), Incompletely Characterized Transport Systems (ICTSs), Channels/Pores, Primary Active Transporters (PATs), and Electrochemical Potential-driven Transporters (EPTs). Numbers of genes belonging to EPTs were highest in the three strains, as 343, 366, and 358, respectively, followed by the number of genes for PATs (255, 260, and 264, respectively) (Fig. 3C). The Major Facilitator Superfamily (MFS) is one of the largest membrane transporter protein super-families, which can facilitate transmembrane transport of solutes such as sugars, drug molecules, peptides, tricarboxylic acid cycle metabolites, and inorganic anions across electrochemical gradients (Pao et al. 1998). ATP-binding cassette (ABC) transporter, as an oversized family of membrane transporter proteins, plays an important role in most organisms (Martinoia et al. 2002). Combined analysis of the results obtained from the TCDB and PHI databases showed that all three tested strains contained more MFS and less ABC. Nearly two-thirds of the ABC family (33 in GXUF-1, 41 in GXUF-2, and 37 in GXUF-3), and more than one-third of the MFS family (53 in GXUF-1, 55 in GXUF-2, and 55 in GXUF-3) transporter proteins were annotated in the PHI database (Fig. 3D), indicating that some membrane transporter proteins may be involved in pathogen-host plant interactions.

Fungal Virulence Factors (DFVF) database is a comprehensive database of known fungal virulence factors. In DFVF database, we annotated 654, 708, and 703 genes for GXUF-1, GXUF-2, and GXUF-3, respectively, none of which exceeded 5% of the predicted genes of the respective strain (Fig. 3A). Notably, the predicted virulence genes orthologous to *HIS3* in *Fusarium* sp. (*g12227.t1*, *g5298.t1*, and *g10686.t1*), *FGA1* in *F. oxysporum* (*g2783.t1, g14953.t1*, and *g4156.t1*), *FGB1* in *F. oxysporum* (*g13786.t1, g12552.t1*, and *g12029.t1*), *FMK1* in *F. oxysporum* (*g4554.t1, g5017.t1*, and *g12914.t1*), and *UBI4* in *Candida albicans* (*g779.t1, g14097.t1*, and *g13971.t1*) showed 100% identity at the amino acid level. Furthermore, *g4523.t1* and *g6373.t1* in GXUF-2 and GXUF-3 were 100% identical to the *FGA2* in *F. oxysporum*, but the predicted ortholog (*g9962.t1*) in GXUF-1 only has 99.7% identity (Table S8). We noticed that the genes predicted by DFVF and PHI in GXUF-1, GXUF-2, and GXUF-3 shared 577, 624, and 620 genes, respectively (Fig. 3D).

**Fig. 4:**
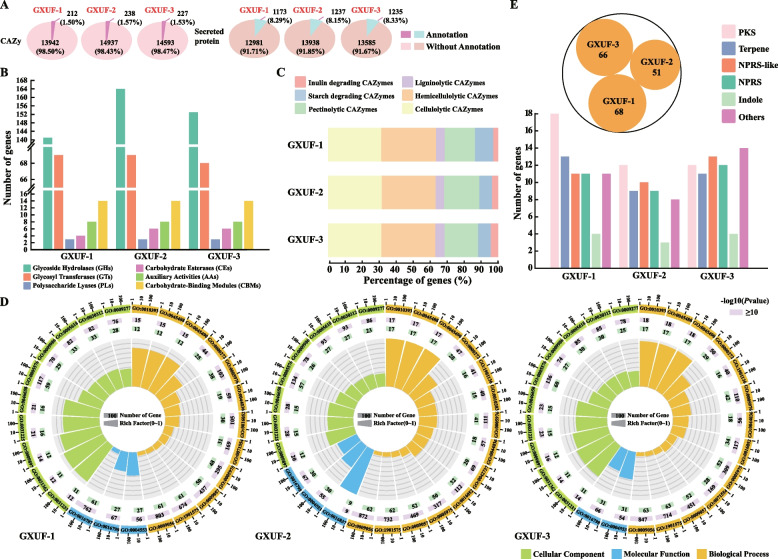
Annotation and analysis of genes using Carbohydrate-Active Enzymes (CAZy) database, as well as analysis of genes encoding Fungal Cell-Wall Degrading Enzymes (FCWDEs), secreted proteins, and the pathways for the biosynthesis of secondary metabolites. **A** The pie chart representing the number of annotated genes of three pathogens causing sugarcane root rot in CAZy database, as well as the number of secreted proteins that the pathogens contained. **B** CAZy functional annotation of the three pathogens causing sugarcane root rot. **C** Comparison of FCWDEs among the three pathogens causing sugarcane root rot. **D** GO enrichment analysis of the genes encoding secreted proteins in these three pathogens causing sugarcane root rot. Numbers in each purple rectangle indicate protein-coding genes in this category. Numbers in each green rectangle indicate secreted protein genes in this category. **E** The core genes involved in the biosynthesis of secondary metabolites in the three pathogens causing sugarcane root rot

**Fig. 5:**
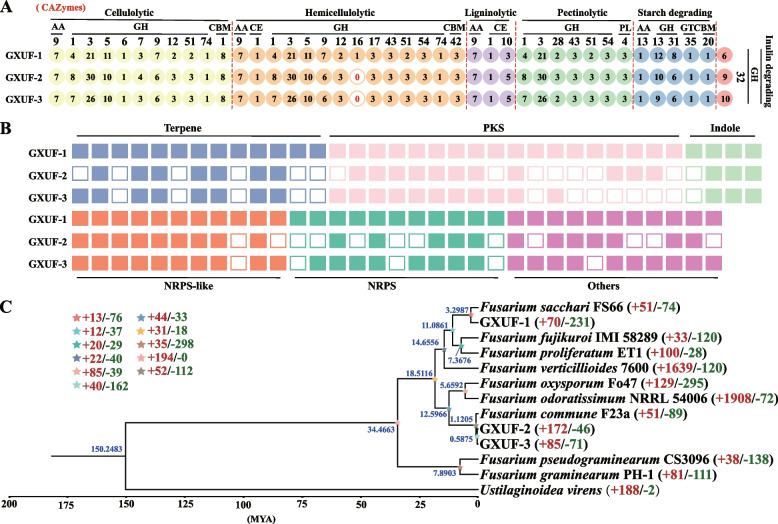
Gene conservation and evolutionary analyses. **A** The types of FCWDEs presented in three pathogens causing sugarcane root rot. The numbers with colored circles represent the number of genes in each pathogen, and colors represent different classifications of FCWDEs. **B** The key genes involved in the secondary metabolism synthesis were analyzed in GXUF-2 and GXUF-3 based on those found in GXUF-1. White squares represent absence, and the different colors represent different key genes of secondary metabolite biosynthesis. **C** Phylogenetic tree of the three *Fusarium* pathogens causing sugarcane root rot and 9 *Fusarium* strains (NCBI published). The phylogenetic tree was constructed based on the whole genome encoding protein sequence. Genome accession: *F*. *sacchari* FS66 (GCA_017165645.1), *F*. *fujikuroi* IMI 58289 (GCA_900079805.1), *F*. *proliferatum* ET1 (GCA_900067095.1), *F*. *verticillioides* 7600 (GCA_000149555.1), *F*. *oxysporum* Fo47 (GCA_013085055.1), *F*. *odoratissimum* NRRL 54006 (GCA_000260195.2), *F*. *commune* F23a (GCA_023065405.1), *F*. *pseudograminearum* CS3096 (GCA_000303195.2), *F*. *graminearum* PH-1 (GCA_000240135.3), *U. virens* (GCA_000687475.1)

Carbohydrate-Active Enzymes (CAZy) is a professional database for prediction of complex carbohydrate-active enzymes. A total of 212, 238, and 227 genes was annotated in the CAZy database for GXUF-1, GXUF-2, and GXUF-3, respectively, accounting for less than 2% of the predicted genes in each strain (Fig. 4A; left panel). Among them, Glycoside Hydrolases (GHs) group had the highest number of genes, as 141, 164, and 153 in GXUF-1, GXUF-2, and GXUF-3, respectively, followed by Glycosyl Transferases (GTs) group with 69, 69, and 68 genes, respectively (Fig. 4B). The three strains contained no more than 20 genes encoding Polysaccharide Lyases (PLs), Carbohydrate Esterases (CEs), Auxiliary Activities (AAs), and Carbohydrate-Binding Modules (CBMs), respectively (Fig. 4B). Based on CAZy annotations, Fungal Cell-Wall Degrading Enzymes (FCWDEs) were classified into cellulolytic CAZymes, hemicellulolytic CAZymes, ligninolytic CAZymes, pectinolytic CAZymes, starch degrading CAZymes, and inulin degrading CAZymes. The proportion of cellulolytic CAZymes and hemicellulolytic CAZymes was relatively higher (more than 63%) than other CAZymes in total FCWDEs, in the three tested strains. In contrast, neither ligninolytic CAZymes nor inulin degrading CAZymes exceeded 6% (Fig. 4C). The detailed classification of FCWDEs of each strain was shown in Fig. 5A.

### Analysis of secreted proteins and secondary metabolite biosynthetic genes

Secreted proteins are highly dynamic and play important roles in cellular homeostasis, immune response, development, proteolysis, adhesion, and extracellular matrix organization (Luo et al. 2012; Stastna and Van Eyk 2012). The putative secreted proteins, which contain signal peptides but no transmembrane domains, were predicted (see Materials and Methods for detailed information). Similar numbers of secreted proteins (1173 for GXUF-1, 1237 for GXUF-2, and 1235 for GXUF-3) were predicted in the genomes of the three tested strains, all of which exceeded 8% of the predicted genes (Fig. 4A; right panel). Functional enrichment analysis showed that organic substance catabolic process (GO:1901575) was the major molecular function in the secreted proteins of the three strains (accounting for about 5% of the total secreted proteins, respectively), and contained a similar number of genes for the catabolic process (GO:0009056), as 61 for GXUF-1, 62 for GXUF-2, and 63 for GXUF-3. Notably, hydrolase activity (GO:0016787) was only enriched in the GXUF-1, which accounted for 5.20% of the total secreted proteins, and it contained the same number of genes (61) as for the organic substance catabolic process (GO:1901575). On the other hand, the drug catabolic process (GO:0042737), cell wall polysaccharide metabolic process (GO:0010383), and carbon-oxygen lyase activity, acting on polysaccharides (GO:0016837) were only enriched in the GXUF-2, which contained 20, 15, and 9 genes, respectively. The number of genes in each category of GO varied in the three tested strains. Carbohydrate metabolic process (GO:0005975) and pectin catabolic process (GO:0045490) found in three pathogens but various in gene numbers (Fig. 4D), which indicates that pectin degradation may play a role in pathogen infestation process.

Generally speaking, secondary metabolites are not essential substances for growth and reproduction of microorganisms, but are often related to microbial pathogenicity (Zotchev 2014). On the other hand, secondary metabolites of microorganisms are an important source of novel bioactive compounds, with potential prospects in drug development (Liu et al. 2019). Identification of secondary metabolic biosynthesis genes and gene clusters, and based on homology analyses (see Materials and Methods for detailed information). 68, 51, and 66 secondary metabolism synthesis-related core genes were respectively identified in the genomes of the three tested strains (Fig. 4E). PKS genes were the most abundant classes of secondary metabolism-related genes among the three strains, with GXUF-1 containing 18, while GXUF-2 and GXUF-3 both containing 12. Indole genes were the least abundant class of secondary metabolism-related genes among the three strains, with none of them exceeding 5 (Fig. 4E). Based on the secondary metabolism core genes in GXUF-1, we searched for common and specific genes among the three tested strains. As shown in Fig. 5B, the common secondary metabolism core genes of the three *Fusarium* strains were 38, including 7 terpene, 8 PKS, 9 NRPS-like, 5 NRPS, 3 indole, and 6 others.

### Evolutionary analysis

We then used the Maximum Likelihood Estimation (MLE) method to assess the genome evolution of the three *Fusarium* strains. GXUF-1 was more closely related to *F. sacchari* FS66, whereas GXUF-2 and GXUF-3 were more closely related to *F. commune* F23a. Fungal species of the same genus clustered closer together but were further away from the outgroup (*Ustilaginoidea virens*) (Fig. 5C). During species evolution, natural selective pressures may lead to expansion and contraction of gene families, and changes in gene families may be related to adaptation to host plants (Demuth and Hahn 2009). Changes in gene families during evolution were further analyzed by estimating the expansion (acquisition) and contraction (loss) of gene families in each branch of the evolutionary tree. Seventy gene families underwent expansion, and 231 gene families underwent contraction in GXUF-1, which is more than the number of expanded and contracted gene families in the *F. sacchari* FS66 strain (51 and 74, respectively). GXUF-2 and GXUF-3 had more expanded gene families (172 and 85, respectively) than *F. commune* F23a strain (51), but less contracted gene families (46 and 71, respectively) than *F commune* F23a (89). These results indicate that among the pathogens causing sugarcane root rot, GXUF-1 lost more genes in the evolution process, while GXUF-2 gained more genes. The number of genes lost and gained in GXUF-3 was comparable in the process of evolution (Fig. 5C).

## Discussion

Multiple fungal pathogens, include *Fusarium* species, *Cylindrocarpon* species, *Phytophthora* species, *Phoma* species, *Rhizoctonia* species, *Alternaria* species, have been reported causing root rot disease to various plant hosts. Among them, *Fusarium* species are a widely distributed group with pathogens that can cause serious diseases (Gao et al. 2015; Zheng et al. 2017). Previous studies have found that *F. sacchari* was a pathogen that infected multiple sites of sugarcane during cultivation, and is responsible for causing sugarcane wilt disease and sugarcane pokkah boeng (Bao et al. 2020; Meng et al. 2020). *F. commune* was isolated and verified as the causal pathogen of sugarcane root rot (Wang et al. 2018; Li et al. 2022). In this study, we confirmed that GXUF-1, GXUF-2, and GXUF-3 were all capable of causing root rot to sugarcane cultivars Badila (chewing cane) and Guitang42 (sugar-making cane). Furthermore, we identified that GXUF-1 as a *F. sacchari* strain, and GXUF-2 and GXUF-3 as *F. commune* strains, based on ANI analysis using the whole-genome sequences of these three strains (Table 1 and Fig. S2A).

In this study, we inoculated the three isolated *Fusarium* strains using 4 reported methods, and analyzed the root rot symptoms and severity to determine a suitable inoculation method for indoor evaluation of *Fusarium* pathogenicity. The result showed that the DWFL method was effective in determining the pathogenicity of *Fusarium* strains to the chewing cane cultivar Badila, as well as sugar-making cane Guitang42 (Table S1-S3, Fig. 2). We infer that it may be because the DWFL method introduces pathogens to the area close to inter-root of the plant, which facilitates the direct interaction between pathogen and plant. Considering that the planting area of Guitang42 is continuously expanding in Guangxi Province, because of its excellent traits such as early maturity, high sugar, high yield, drought resistance, lodging resistance, suitable for machine harvesting, and wide adaptability (Wang et al. 2015), the ability of the three *Fusarium* strains to infect and cause root rot in Guitang42 pose a potential risk in future sugarcane plantation. Thus, it is very important to strengthen inspection and quarantine, disease detection, and comprehensive prevention, and control of sugarcane root rot.

We found that the genome size of GXUF-1 (44,739,610 bp) was slightly smaller than that of the *F. sacchari* strain (45,739,938 bp) causing banana leaf blight (Cui et al. 2021), whereas the genome sizes (47,303,747 and 48,236,101 bp) of GXUF-2 and GXUF-3 were slightly larger than that of the *F. commune* strain causing lotus rhizome rot (46,211,149 bp) (Table 1) (Kuang et al. 2022). All strains were approximately in the same order of magnitude but slightly different in size, which may be related to sequencing technology and depth (Eastman and Yuan 2015; Mao and Chen 2018), and likewise it could be due to changes in biological functions in similar species (Grilli et al. 2014). It is peculiar that DNA transposon sequences were predicted in the genomes of GXUF-2 and GXUF-3 (*F. commune*), while not in GXUF-1 (*F. sacchari*) genome (Table 2). At present the published *F. sacchari* genome sequence (str. FS66) (Cui et al. 2021) did not provide information on (if any) types or frequency of transposon sequences, although transposons were widely distributed in genome of *Fusarium* spp. (Purayil et al. 2023), including GXUF-2 and GXUF-3 sequenced in this study. The possible reason, we infer could be due to the limitation of the second-generation sequencing on long repeat sequences (Massip et al. 2015). We believe that with the help of the third-generation sequencing protocol, more repetitive sequences (transposons) can be effectively assembled.

The coding genes of these three strains could be annotated by multiple databases, and some of their genes could be individually annotated in different public databases. According to the annotation results of the KOG, GO, and KEGG databases, the gene functions of the three *Fusarium* strains are mostly concentrated in carbohydrate transport and metabolism (Fig. S3), cellular process (Table S5), and carbohydrate metabolism (Fig. S5). Furthermore, the NR database annotation revealed that GXUF-1 was genetically close to *F. proliferatum*, which could cause root rot on soybean (*Glycine max*) (Díaz Arias et al. 2011), while GXUF-2 and GXUF-3 had a high genetic identity with *F. oxysporum* f. sp. *lycopersici* 4287 (Fig. S8A), which could cause tomato wilt disease (Ma et al. 2010). The potential pathogenic genes could be identified based on annotation using proprietary databases (such as PHI, DFVF, CAZy, etc.), secreted proteins, and secondary metabolite biosynthetic genes (Fig. 3-5). The genes of the categories of increased pathogenicity (hypervirulence) and effector (plant avirulence determinant) annotated in the PHI database (Table S7) could be considered as potential pathogenic genes. Furthermore, these three *Fusarium* strains had similar numbers of the predicted CAZy genes (Fig. 4A), and the FCWDEs were classified based on the annotations in the CAZy database (Fig. 4C). Given that *Fusarium* species are soil-borne pathogens requiring FCWDEs for fungal colonization on plant tissues (Ye et al. 2011), as expected, these three *Fusarium* strains possess abundant pectinolytic, cellulolytic, and hemicellulolytic CAZymes related genes (Fig. 5A). It has been reported that the polygalacturonase is a class of glycoside hydrolases (GH28) playing a key role in pectinase degradation (Zhao et al. 2014). We found that all these three *Fusarium* strains have the pectinolytic CAZymes (Fig. 5A), which potentially contributing to decomposing plant cell walls and causing sugarcane disease.

Based on the evolutionary analysis of the whole-genome sequences of these three *Fusarium* strains, we found that GXUF-1 was homologous to *F. sacchari* FS66, and GXUF-2 and GXUF-3 were extremely homologous to *F. commune* F23a (Fig. 5C). Notably, three *Fusarium* strains had experienced the gene contraction (loss) during evolution process, which is often overlooked as an evolutionary driver, mainly because it is associated with the absence of redundant gene duplicates without obvious functional consequences (Olson 1999). However, a growing body of genomic data suggests that gene loss is an obvious source of genetic variation that may underlie phenotypic diversity (Albalat and Cañestro 2016), which may account for the difference in colony morphology and pathogenicity of GXUF-2 and GXUF-3, despite both of them belong to *F. commune.*

In summary, we confirmed that three *Fusarium* strains are able to cause sugarcane root rot to chewing cane and sugar-making cane cultivars. Due to the transmission and epidemiological pattern of *Fusarium* pathogens, there is a risk that it may evolve into a mainstream disease in sugarcane (Ren et al. 2021). Future research is necessary on the epidemiological pattern of occurrence and the mechanism of monitoring, prevention, and control sugarcane root rot. Furthermore, we also performed whole-genome sequencing and pathogen evolutionary analysis of these three strains, providing a theoretical basis for exploring the pathogenic mechanisms of *Fusarium* pathogens, which may be beneficial to the effective interruption, prevention and control sugarcane root rot.

## Materials and methods

### Isolation, morphological observation and pathogenicity test of the three *Fusarium* strains

The diseased plants with typical symptoms of sugarcane root rot were collected and separated according to the tissue separation method (Xiao et al. 2020). Diseased tissue of 3–5 mm was excised from the junction of the diseased and healthy roots of the diseased plants, treated with 75% alcohol for 30 s and subsequently with 2% sodium hypochlorite solution for 2 min, washed with sterile water for 3 times, and then placed on PDA medium (Fig. S1A), for culturing at 28 °C for 3 d. Single colonies of fungi were picked for purification and culture.

The fungal hyphae and spore were observed under a microscope and classified according to *The Fusarium laboratory manual* (Leslie and Summerell 2006). The pathogen(s) was/were verified according to Koch’s postulates. The isolated pathogenic fungal strains that potentially cause sugarcane root rot were cultured in 100 mL Potato Dextrose Broth (PDB) medium at 28 °C and shaking at 180 r/min for 5 days. The spores were collected and diluted with sterile water to reach a concentration of 1 × 10^6^ spores/mL. The fungal suspension was then inoculated separately into sterile soil containing healthy sugarcane tubers of equal size (to avoid unequal residual microbes brought in by the tubers). After the disease occurred, the diseased root system was re-isolated and cultured, and the isolated strain(s) was/were identified to observe whether the properties of the inoculated strain(s) were consistent.

### Effect of different strains and inoculation methods on sugarcane (chewing cane) phenotype and rhizosphere soil physicochemical properties

The healthy sugarcane (Badila) tubers of equal size were selected and disinfected by immersing in 1% sodium hypochlorite (to avoid unequal residual microbes brought in by the tubers) for later use. The isolated pathogenic fungal strains causing sugarcane root rot were cultured in PDB or on PDA media, respectively. The spores of pathogens cultured in 100 mL of PDB medium at 28 °C and shaken at 180 r/min for 5 d were collected and diluted with sterile water to reach a concentration of 1 × 10^6^ spores/mL. These fungal suspensions were inoculated to the healthy sugarcane tubers according to DWFL and FLMWS methods, respectively (Fig. S1B). Furthermore, fungal discs with pathogens cultured on PDA plate at 28 °C were split into multiple 5-mm and inoculated to the healthy sugarcane tubers according to DIFC and FCMWS methods, respectively (Fig. S1B). Three repeats were performed for each treatment, and sugarcane without inoculated pathogens as the blank control.

After 45 days of sugarcane growth under different inoculation methods of the various fungal pathogens, we measured the aboveground plant height, fresh weight, and dry weight. Furthermore, the roots of each sugarcane were dug out and loosely attached soil was removed by manual shaking, whereas the rhizosphere soil was collected from the surface of the roots and sealed in sterile ziplock bags for later use. The sugarcane roots were washed with clean water and photographed. The total root length, total root surface area, and total root volume were measured using the software RhizoVision Explorer V2.0.2 (Seethepalli et al. 2021).

To assess the differences in the physicochemical properties of rhizosphere soil, the soil samples were air dried at room temperature (25–28 °C) and passed through a 2 mm sieve to remove stones and plant residues. The soil TC and TN were determined using the element analyzer (Thermo Scientific). The soil AP was measured by the sodium bicarbonate extraction molybdenum antimony anti-colorimetric method (Olsen et al. 1954) and the AK was quantified by ammonium acetate extraction-flame photometry (Mc Lean and Watson 1985). The contents of Ca, Mg, Cu, Mn, and Fe were extracted using the Mehlich-III procedure and determined by atomic absorption spectrophotometry (Mehlich 1984).

### Pathogenicity assay of *Fusarium* strains on sugar-making cultivar Guitang42 using DWFL method

Based on the study on the effects of various inoculation methods on sugarcane (chewing cane) phenotype and rhizosphere soil physicochemical properties, the most suitable method for inoculating pathogens on sugarcane (making sugar) tissue culture seedlings was identified. The virus-free tissue culture seedlings (Guitang42) were carefully extracted from the tissue culture flask after refining, ensuring that the original roots were retained. The residual medium was thoroughly washed off with pure water, and the seedlings were then transplanted into a nutrition cup to establish root growth for further use. The pathogens causing sugarcane root rot were inoculated on sugarcane (making sugar) using the suitable inoculation (DWFL) method. After 45 days, the plant height, dry weight, and fresh weight of the aerial part were measured. The RhizoVision Explorer V2.0.2 root analysis system software was used to analyze the total root length, total root surface area, and total volume of the scanned images. All statistical analyses were performed using SPSS V27 software (IBM, USA), and determined using analysis of variance (ANOVA) followed by Duncan’s test. A *P* value < 0.05 was considered statistically significant.

### Whole-genome sequencing and annotation the pathogens causing sugarcane root rot

The whole genomic DNA of pathogens causing sugarcane root rot was extracted by the modified cetyltrimethylammonium bromide (CTAB) methods (Allen et al. 2006). The concentration of DNA in each sample was quantified with Qubit® dsDNA HS assay kit (Invitrogen), while the quality was assessed by gel electrophoresis with 1% agarose. Genomic DNA was fragmented using an M220 Focused-ultrasonicator (Covaris, Massachusetts), and sequencing library construction was conducted with VAHTSTM Universal DNA Library Prep kit for Illumina. The library was quality-checked by Qsep-400 and the library concentration was quantified using Qubit 3.0 to check if the quality of the library meets with the following standards: concentration ≥ 1 ng/μL, the center value of fragment 430–530 bp, average value 420–580 bp, normal distribution of peak pattern, single fragment without spurious peak. After the library was qualified, Illumina NovaSeq6000 (San Diego) was used for on-machine sequencing. The whole-genome sequencing data and genome assemblies were deposited in the SRA (SRX22139249 for GXUF-1, SRX22139431 for GXUF-2, and SRX22139432 for GXUF-3), and assembly (GCA_033675115.1 for GXUF-1, GCA_033782975.1 for GXUF-2, and GCA_033782995.1 for GXUF-3) at NCBI BioProject PRJNA1029320 (GXUF-1) and PRJNA1029782 (GXUF-2 and GXUF-3).

Before assembly, sequencing raw datum from each sample was subjected to quality control using Fastp V0.20.0 to clear out the sequencing adapter and low-quality reads (Chen et al. 2018). De novo whole-genome assembly of the pathogens causing sugarcane root rot was performed using Megahit V1.2.9 at default mode (Li et al. 2015). BUSCO was generally used in the evaluation of the completeness of a genome assembly, we applied BUSCO V5.4.6 to assess the quality of whole-genome assembly in our study (Manni et al. 2021). ANI was calculated using the ANI calculator (Yoon et al. 2017). Based on the constructed repeat sequence database, the repeat sequence de novo prediction was performed on the assembly results using RepeatModeler V2.0.3, and then RepeatMasker V4.1.4 was used to find the position and frequency of various types of repeat sequences on the genome segment (Tarailo-Graovac and Chen 2009). Gene structure and protein coding gene models were predicted using the Augustus V3.4.0 program (Stanke et al. 2006). The non-coding RNAs were identified by employing Infernal V1.1.2 software (Nawrocki and Eddy 2013) to search against the Rfam database (http://rfam.xfam.org/). Pseudogene prediction was conducted using the pipeline V2.00 software (Zhong et al. 2022).

To obtain comprehensive information on gene function, gene functional annotation was carried out on the sequences, mainly based on the databases including NR, KOG, GO, KEGG, Pfam (the emapper V1.0.3 annotation tool by eggNOG) (Huerta-Cepas et al. 2017), Swiss-Prot (Uniprot Consortium 2013), P-450 (http://drnelson.uthsc.edu/CytochromeP450.html), TCDB (Saier et al. 2016), CAZy (Cantarel et al. 2009), and FCWDEs (putative FCWDEs were identified and classified based on the CAZy results). Secondly, PHI (Urban et al. 2014) and DFVF database (Lu et al. 2012) were used to find pathogenicity and virulence-related genes. Furthermore, the signal peptide was predicted with SignalP V6.0 (Teufel et al. 2022) and transmembrane domains were predicted using TMHMM (https://services.healthtech.dtu.dk/services/TMHMM-2.0/). Combining the results obtained from these two databases, we considered the proteins with the presence of signal peptide but no transmembrane domain as putatively secreted proteins. Finally, antiSMASH V7.0.0 software (https://fungismash.secondarymetabolites.org/) was used to identify secondary metabolite biosynthetic genes and gene clusters based on Hidden Markov Models of the specified types, and based on homology analyses, the secondary metabolic core genes were analyzed by BLAST for their presence in presence and absence in the genomes.

### Evolutionary analysis

The whole-genome sequence data of the strains used for evolutionary analysis were downloaded from the NCBI Genome database (https://www.ncbi.nlm.nih.gov/genome). The default parameters of OrthoFinder V2.5.5 software (Emms and Kelly 2019) was used for identifying homologous gene families when we input the whole genome encoded protein sequences of *Fusarium* species, including the pathogens causing sugarcane root rot, in this study. Based on the results of homologous gene family clustering analysis, single copies of homologous genes were selected for multiple sequence alignment using Muscle V5.1 (Edgar 2022), and then a phylogenetic tree was constructed using the MLE method thorough RAxML V8.2.12 software (Stamatakis 2014). The divergence time was estimated by using the program Mcmctree V4.10.0 (Yang 2007), which was part of the PAML package. We measured the expansion and contraction of orthologous gene families based on a maximum likelihood tree using CAFÉ V5.0.0 software (De Bie et al. 2006).

### Supplementary Information

**Additional file 1: Fig. S1.** Experimental design schemes. **Fig. S2.** Average Nucleotide Identity (ANI) analysis, gene length and annotation. **Fig. S3.** Functional annotation based on the KOG classification. **Fig. S4.** Analysis of GO categories. **Fig. S5.** The KEGG metabolic pathway classification diagram. **Fig. S6.** Genes detected in KEGG map of tryptophan metabolism (00380). **Fig. S7.** Genes detected in KEGG map of MAPK signaling pathway (04010). **Fig. S8.** Statistics of gene annotation using general databases.

**Additional file 2: Table S1.** Effect of different strains and inoculation methods on aboveground phenotypes of sugarcane (Badila). **Table S2.** Effect of different strains and inoculation methods on underground phenotypes of sugarcane (Badila). **Table S3.** Effect of different strains and inoculation methods on rhizosphere soil physicochemical properties. **Table S4.** The quality of all whole-genome sequencing samples before and after filtration using Fastp. **Table S5.** Functional classification of Gene Ontology (GO) categories. **Table S6.** Detailed Kyoto Encyclopedia of Genes and Genomes (KEGG) pathway annotation of three tested strains. **Table S7.** Pathogen-Host Interactions (PHI) database annotated genes belonging to increased pathogenicity (hypervirulence) and effector (plant avirulence determinant) in the three tested strains. **Table S8.** Comparison of the predicted virulence genes in three tested strains.

## Data Availability

The whole-genome sequencing data and genome assemblies were deposited in the SRA (SRX22139249 for GXUF-1, SRX22139431 for GXUF-2, and SRX22139432 for GXUF-3), and assembly (GCA_033675115.1 for GXUF-1, GCA_033782975.1 for GXUF-2, and GCA_033782995.1 for GXUF-3) at NCBI BioProject PRJNA1029320 (GXUF-1) and PRJNA1029782 (GXUF-2 and GXUF-3). Other data that support the findings of this study have been provided in the supplementary information files.

## Abbreviations

rDNA-ITS: rDNA internal transcribed spacer
EF-1alpha: Elongation factor 1-alpha
DWFL: Direct watering fungal liquid
FLMWS: Fungal liquid mixed with soil
DIFC: Direct inoculation fungal cultures
FCMWS: Fungal cultures mixed with soil
RS: Root soaking
PDA: Potato Dextrose Agar
CMC: Carboxymethyl cellulose
TC: Total carbon
TN: Total nitrogen
AP: Available phosphorus
AK: Available potassium
Ca: Calcium
Mg: Magnesium
Mn: Manganese
Fe: Iron
Cu: Copper
BUSCO: Benchmarking Universal Single-Copy Orthologs
ANI: Average Nucleotide Identity
LTRs: Long terminal repeats
LINEs/SINEs: Long/Short interspersed nuclear elements
RCs: Rolling circles
tRNAs: transfer RNAs
rRNAs: ribosomal RNAs
sRNAs: small RNAs
snRNAs: small nucleolar RNAs
miRNAs: microRNAs
KOG: Eukaryotic Orthologous Groups
GO: Gene Ontology
BP: Biological Process
MF: Molecular Function
CC: Cellular Component
KEGG: Kyoto Encyclopedia of Genes and Genomes
CP: Cellular Processes
EIP: Environmental Information Processing
GIP: Genetic Information Processing
HD: Human Diseases
M: Metabolism
OS: Organismal Systems
NR: Non-Redundant protein sequence
Pfam: Protein families database of alignments and hidden Markov models
P-450: Cytochrome P-450
PHI: Pathogen-Host Interactions
TCDB: Transporter Classification Database
TECs: Transmembrane Electron Carriers
GTs: Group Translocators
AFIT: Accessory Factors Involved in Transport
ICTSs: Incompletely Characterized Transport Systems
PATs: Primary Active Transporters
EPTs: Electrochemical Potential-driven Transporters
MFS: Major Facilitator Superfamily
ABC: ATP-binding cassette
DFVF: Fungal Virulence Factor
CAZy: Carbohydrate-Active Enzymes
GHs: Glycoside Hydrolases
GTs: Glycosyl Transferases
PLs: Polysaccharide Lyases
CEs: Carbohydrate Esterases
AAs: Auxiliary Activities
CBMs: Carbohydrate-Binding Modules
FCWDEs: Fungal Cell-Wall Degrading Enzymes
MLE: Maximum Likelihood Estimation
PDB: Potato Dextrose Broth
CTAB: Modified cetyltrimethylammonium bromide
