# Supplementary material for: Whole-genome sequencing of Fusarium spp. causing sugarcane root rot on both chewing cane and sugar-making cane
Source: Stress Biol. 2024 Jan 25;4(1):7. doi: 10.1007/s44154-023-00145-7 (PMC10811303; doi:10.1007/s44154-023-00145-7)
Supplement: Supplementary file 1 — Additional file 1: Fig. S1. Experimental design schemes. Fig. S2. Average Nucleotide Identity (ANI) analysis, gene length and annotation. Fig. S3. Functional annotation based on the KOG classification. Fig. S4. Analysis of GO categories. Fig. S5. The KEGG metabolic pathway classification diagram. Fig. S6. Genes detected in KEGG map of tryptophan metabolism (00380). Fig. S7. Genes detected in KEGG map of MAPK signaling pathway (04010). Fig. S8. Statistics of gene annotation using general databases. [file 44154_2023_145_MOESM1_ESM.pdf]

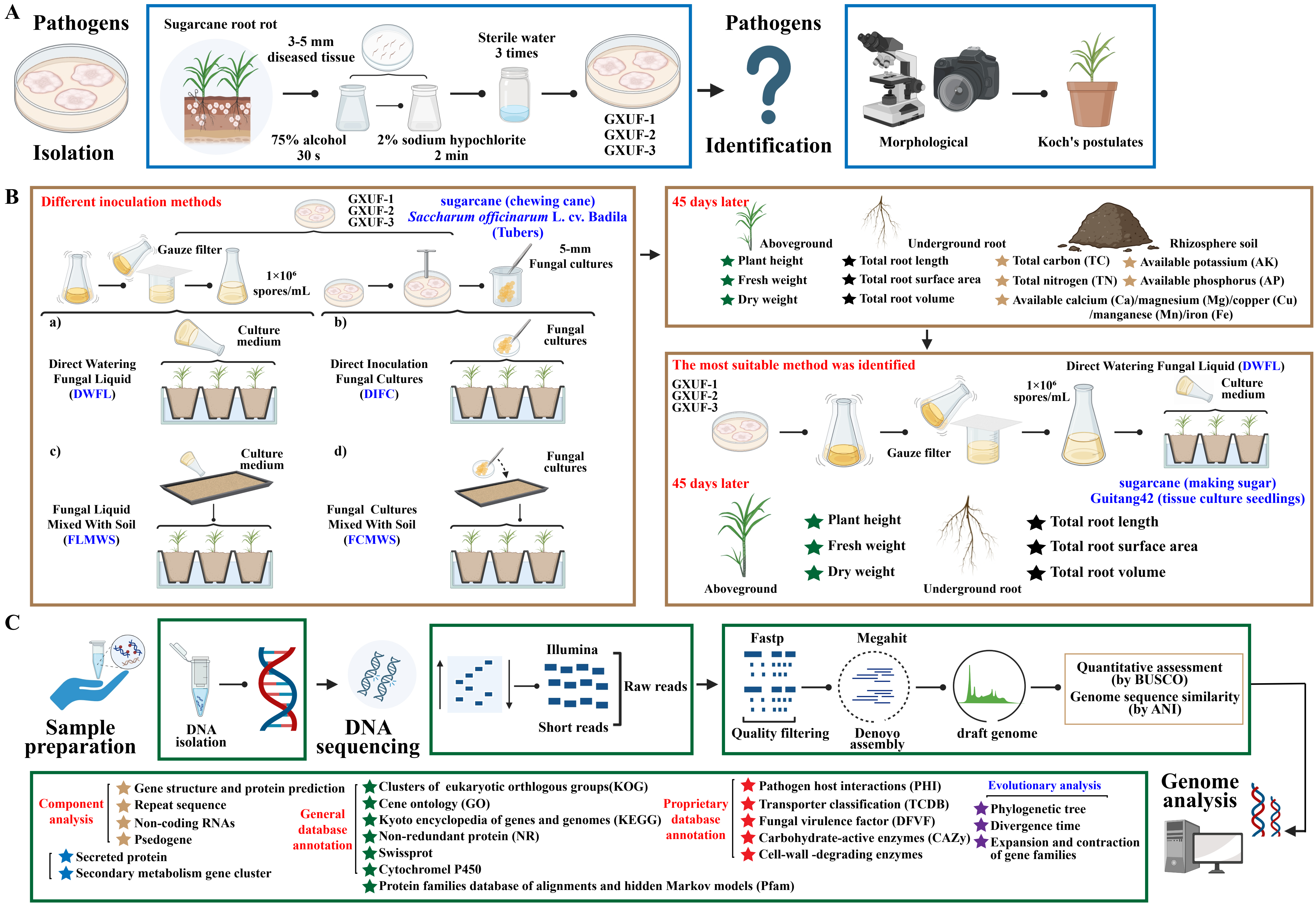
A
Pathogens
Isolation
Sugarcane root rot
3-5 mm
diseased tissue
Sterile water
3 times
75% alcohol
30 s
2% sodium hypochlorite
2 min
GXUF-1
GXUF-2
GXUF-3
Pathogens
Identification
Morphological
Koch's postulates
B
Different inoculation methods
GXUF-1
GXUF-2
GXUF-3
sugarcane (chewing cane)
Saccharum officinarum L. cv. Badila
(Tubers)
Gauze filter
1×10⁶
spores/mL
5-mm
Fungal cultures
a)
Culture
medium
Direct Watering
Fungal Liquid
(DWFL)
b)
Fungal
cultures
Direct Inoculation
Fungal Cultures
(DIFC)
c)
Culture
medium
Fungal Liquid
Mixed With Soil
(FLMWS)
d)
Fungal
cultures
Fungal Cultures
Mixed With Soil
(FCMWS)
45 days later
Aboveground
Plant height
Fresh weight
Dry weight
Underground root
Total root length
Total root surface area
Total root volume
Rhizosphere soil
Total carbon (TC)
Total nitrogen (TN)
Available potassium (AK)
Available phosphorus (AP)
Available calcium (Ca)/magnesium (Mg)/copper (Cu)
/manganese (Mn)/iron (Fe)
The most suitable method was identified
Direct Watering Fungal Liquid (DWFL)
GXUF-1
GXUF-2
GXUF-3
1×10⁶
spores/mL
Culture
medium
Gauze filter
45 days later
sugarcane (making sugar)
Guitang42 (tissue culture seedlings)
Plant height
Fresh weight
Dry weight
Total root length
Total root surface area
Total root volume
Aboveground
Underground root
C
Sample
preparation
DNA
isolation
DNA
sequencing
Illumina
Raw reads
Short reads
Fastp
Megahit
Quality filtering
Denovo
assembly
draft genome
Quantitative assessment
(by BUSCO)
Genome sequence similarity
(by ANI)
Genome
analysis
Component
analysis
Gene structure and protein prediction
Repeat sequence
Non-coding RNAs
Psedogene
Secreted protein
Secondary metabolism gene cluster
General
database
annotation
Clusters of eukaryotic orthlogous groups(KOG)
Cene ontology (GO)
Kyoto encyclopedia of genes and genomes (KEGG)
Non-redundant protein (NR)
Swissprot
Cytochromel P450
Protein families database of alignments and hidden Markov models (Pfam)
Proprietary
database
annotation
Pathogen host interactions (PHI)
Transporter classification (TCDB)
Fungal virulence factor (DFVF)
Carbohydrate-active enzymes (CAZy)
Cell-wall -degrading enzymes
Evolutionary analysis
Phylogenetic tree
Divergence time
Expansion and contraction
of gene families

Fig. S1 Experimental design schemes.
A Isolation and identification of sugarcane root rot pathogen(s). **B** Using two factors (strain and inoculation method) to process fruit cane cultivar Badila tubers, assessment of the most suitable inoculation method based on plant phenotype and rhizosphere soil physicochemical properties, and then with this method for the pathogenicity assay of *Fusarium* strains on sugar-making cultivar Guitang42. **C** Whole-genome sequencing and evolutionary analysis of three *Fusarium* pathogens causing sugarcane root rot. Some images were created using BioRender.com.

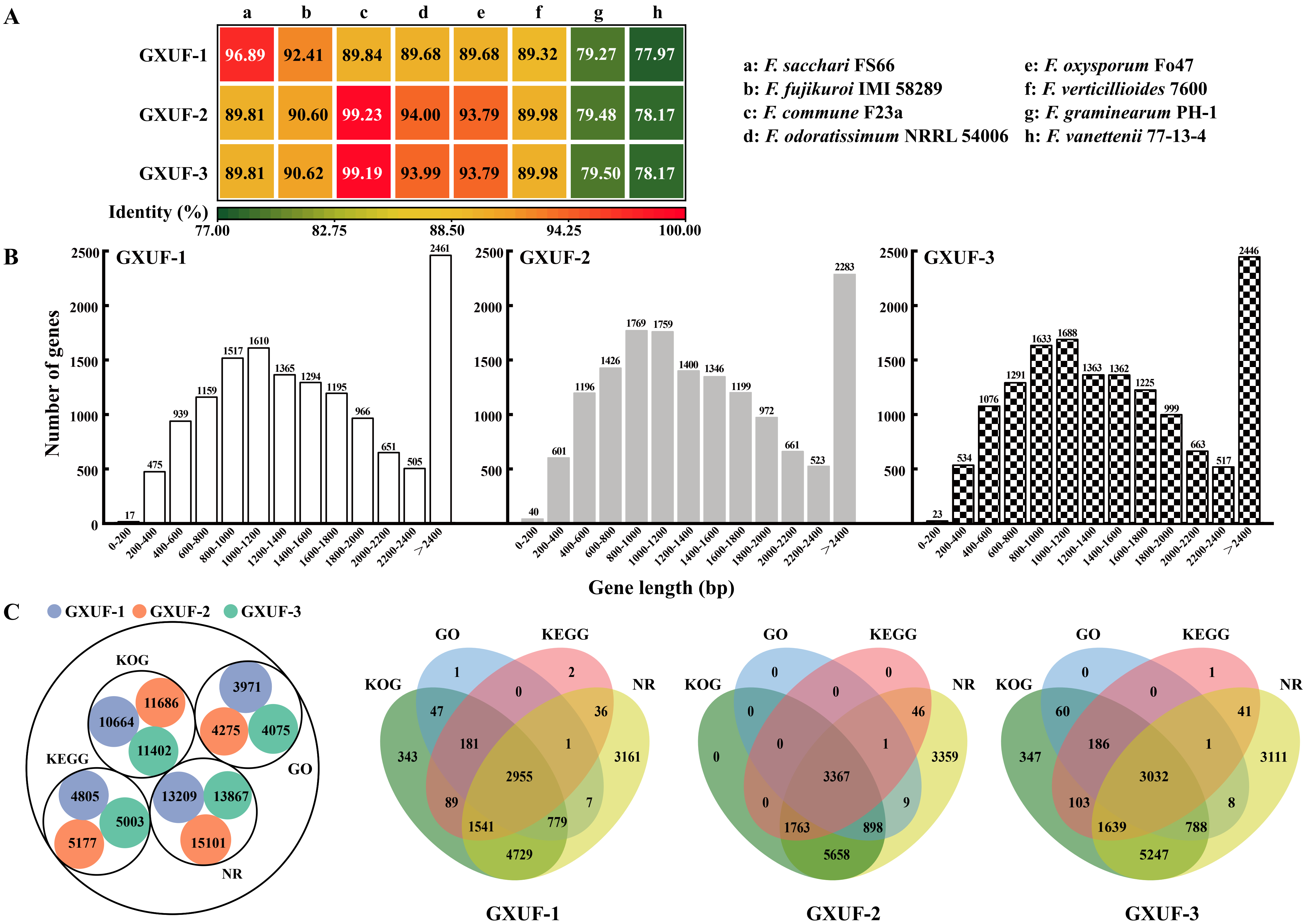

A
a b c d e f g h
GXUF-1 96.89 92.41 89.84 89.68 89.68 89.32 79.27 77.97
GXUF-2 89.81 90.60 99.23 94.00 93.79 89.98 79.48 78.17
GXUF-3 89.81 90.62 99.19 93.99 93.79 89.98 79.50 78.17
Identity (%)
77.00 82.75 88.50 94.25 100.00
a: F. sacchari FS66
b: F. fujikuroi IMI 58289
c: F. commune F23a
d: F. odoratissimum NRRL 54006
e: F. oxysporum Fo47
f: F. verticillioides 7600
g: F. graminearum PH-1
h: F. vanettenii 77-13-4
B
GXUF-1
GXUF-2
GXUF-3
Number of genes
Gene length (bp)
0-200 200-400 400-600 600-800 800-1000 1000-1200 1200-1400 1400-1600 1600-1800 1800-2000 2000-2200 2200-2400 >2400
GXUF-1: 17 475 939 1159 1517 1610 1365 1294 1195 966 651 505 2461
GXUF-2: 40 601 1196 1426 1769 1759 1400 1346 1199 972 661 523 2283
GXUF-3: 23 534 1076 1291 1633 1688 1363 1362 1225 999 663 517 2446
C
GXUF-1 GXUF-2 GXUF-3
KOG 10664 11686 11402
GO 3971 4275 4075
KEGG 4805 5177 5003
NR 13209 15101 13867
GO KEGG KOG NR
GXUF-1: 1 2 47 0 36 343 181 1 3161 2955 89 7 1541 779 4729
GXUF-2: 0 0 0 0 46 0 0 1 3359 3367 0 9 1763 898 5658
GXUF-3: 0 1 60 0 41 347 186 1 3111 3032 103 8 1639 788 5247

Fig. S2 Average Nucleotide Identity (ANI) analysis, gene length and annotation.

A The whole genome ANI analysis of three pathogens causing sugarcane root rot. Reference genome: *F. sacchari* FS66 (GCA_017165645.1), *F. fujikuroi* IMI 58289 (GCA_900079805.1), *F. commune* F23a (GCA_023065405.1), *F. odoratissimum* NRRL 54006 (GCA_000260195.2), *F. oxysporum* Fo47 (GCA_013085055.1), *F. verticillioides* 7600 (GCA_000149555.1), *F. graminearum* PH-1 (GCA_000240135.3), *F. vanettenii* 77-13-4 (GCA_000151355.1). **B** Distribution diagram of gene lengths of three pathogens causing sugarcane root rot. **C** Circular packing representing the number of annotated genes in the whole genome of the pathogens causing sugarcane root rot in the Non-Redundant protein sequence (NR), Eukaryotic Orthologous Groups (KOG), Gene Ontology (GO), and Kyoto Encyclopedia of Genes and Genomes (KEGG), respectively. Venn diagrams indicating the common and unique genes among four major databases (NR, KOG, GO and KEGG).

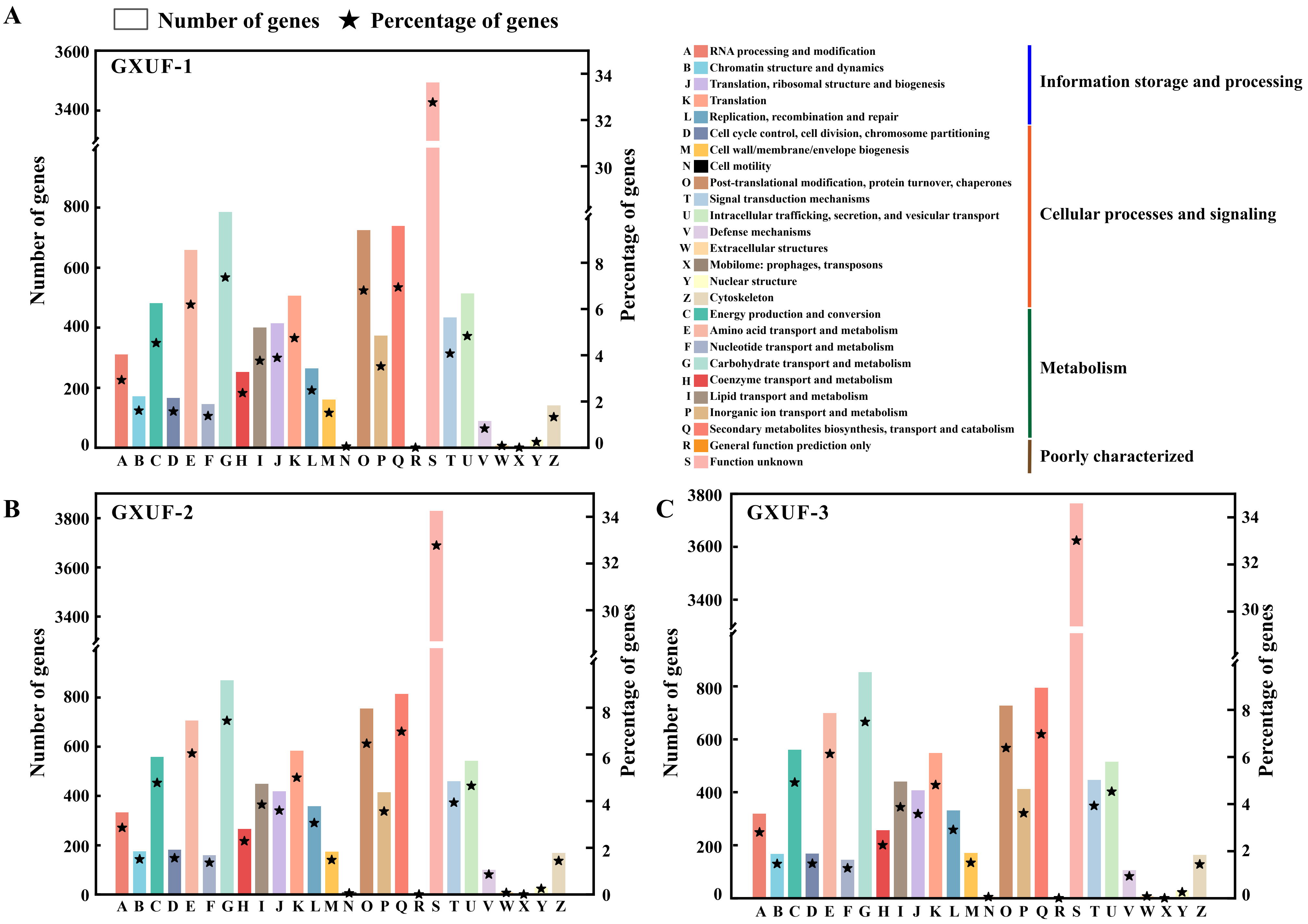

A
Number of genes
Percentage of genes
GXUF-1
Number of genes
Percentage of genes
A RNA processing and modification
B Chromatin structure and dynamics
J Translation, ribosomal structure and biogenesis
K Translation
L Replication, recombination and repair
D Cell cycle control, cell division, chromosome partitioning
M Cell wall/membrane/envelope biogenesis
N Cell motility
O Post-translational modification, protein turnover, chaperones
T Signal transduction mechanisms
U Intracellular trafficking, secretion, and vesicular transport
V Defense mechanisms
W Extracellular structures
X Mobilome: prophages, transposons
Y Nuclear structure
Z Cytoskeleton
C Energy production and conversion
E Amino acid transport and metabolism
F Nucleotide transport and metabolism
G Carbohydrate transport and metabolism
H Coenzyme transport and metabolism
I Lipid transport and metabolism
P Inorganic ion transport and metabolism
Q Secondary metabolites biosynthesis, transport and catabolism
R General function prediction only
S Function unknown
Information storage and processing
Cellular processes and signaling
Metabolism
Poorly characterized
B
GXUF-2
Number of genes
Percentage of genes
C
GXUF-3
Number of genes
Percentage of genes
A B C D E F G H I J K L M N O P Q R S T U V W X Y Z

Fig. S3 Functional annotation based on the KOG classification.
A GXUF-1; **B** GXUF-2; **C** GXUF-3.

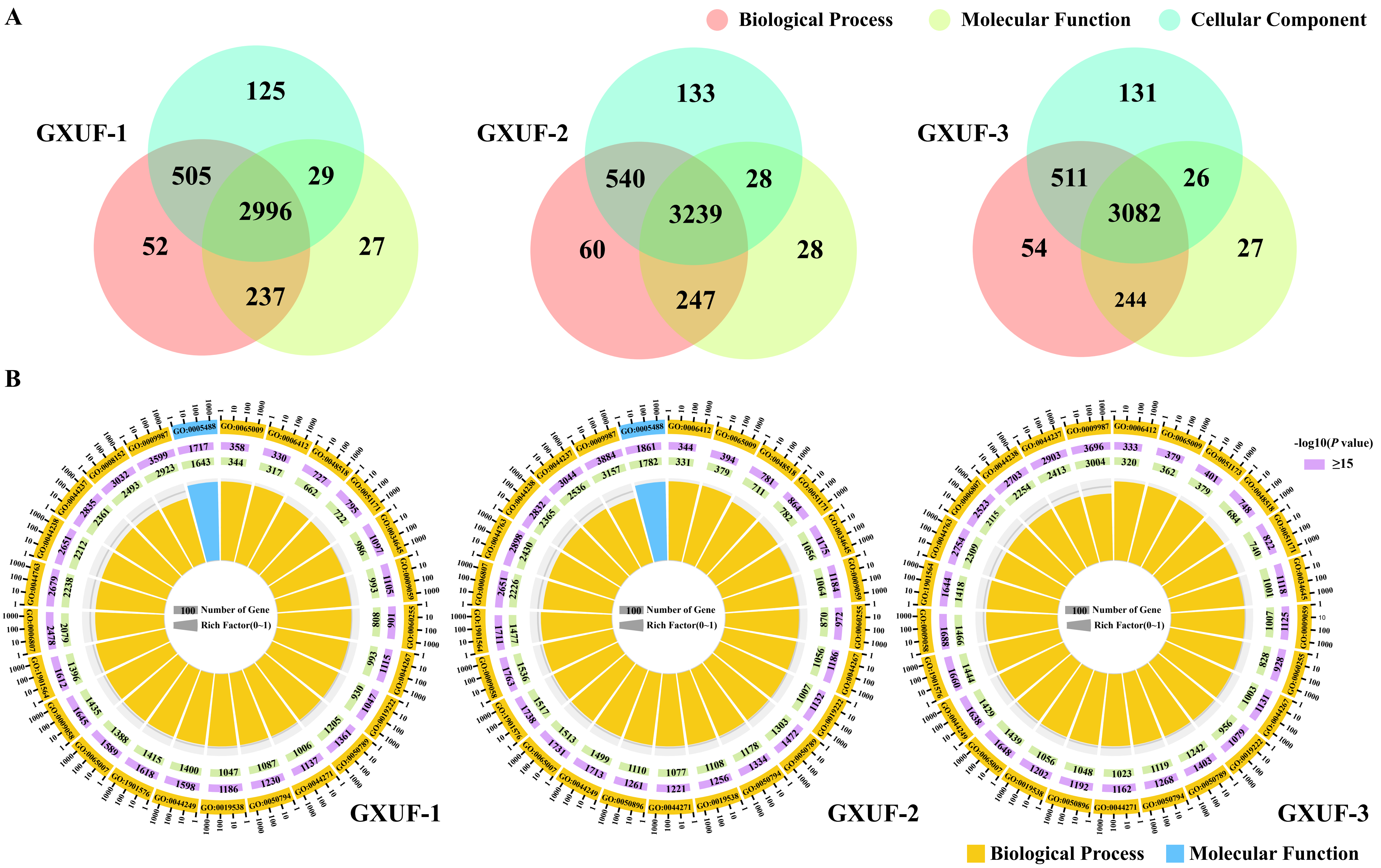
A
Biological Process
Molecular Function
Cellular Component
GXUF-1
125
505
29
2996
52
27
237
GXUF-2
133
540
28
3239
60
28
247
GXUF-3
131
511
26
3082
54
27
244
B
-log10(P value)
≥15
100 Number of Gene
Rich Factor(0~1)
GXUF-1
GXUF-2
GXUF-3
Biological Process
Molecular Function

Fig. S4 Analysis of GO categories.

A The GO categories of three pathogens causing sugarcane root rot were analyzed for shared genes. **B** GO functional enrichment analysis visualized with an enrichment circle diagram (The protein-coding genes of the respective strains were used as background genes, together with the shared genes as target genes). Numbers with purple rectangle indicate protein-coding genes in this category. Numbers with green rectangle indicate shared genes under GO category in this category.

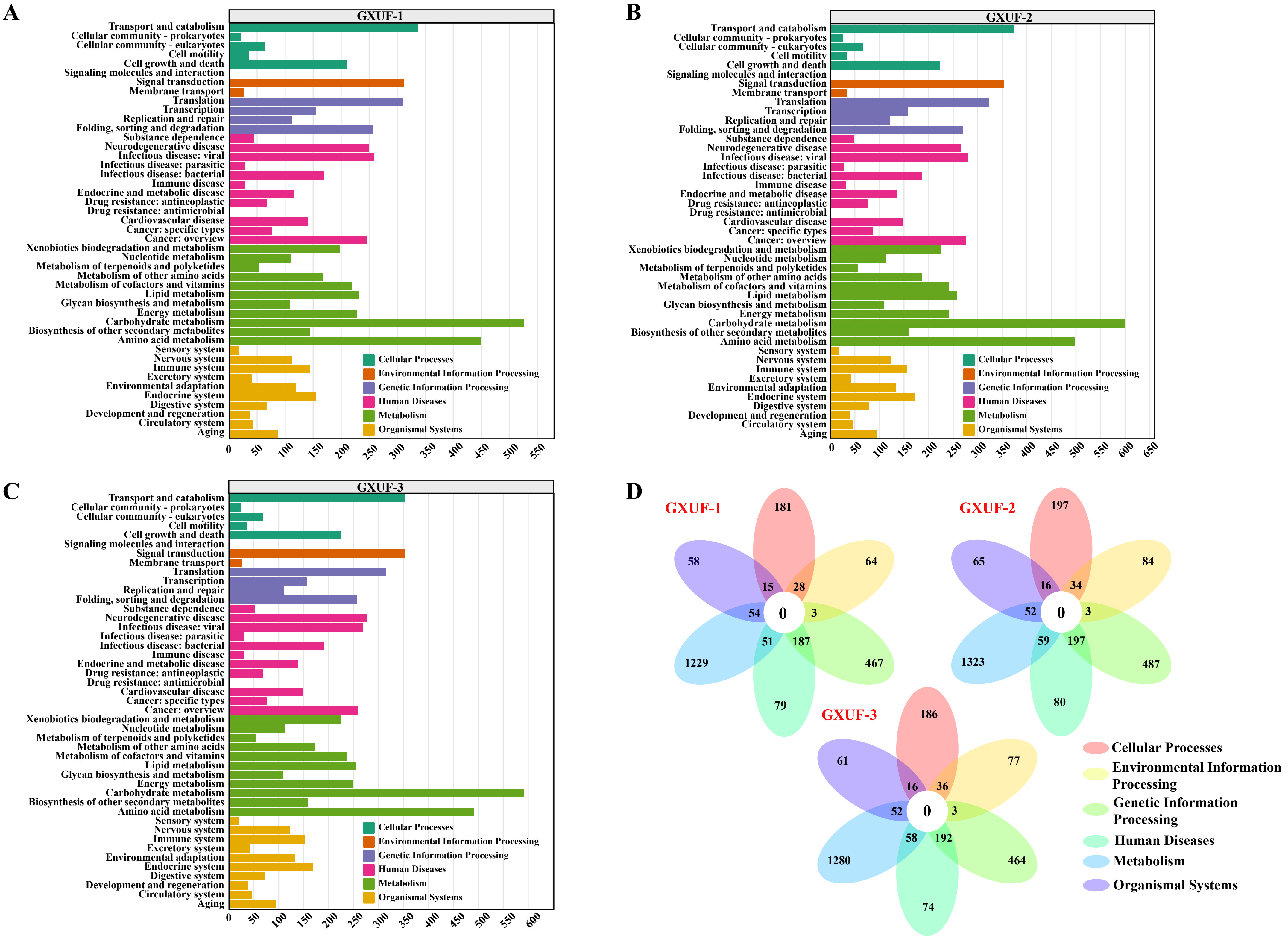

A
GXUF-1
Transport and catabolism
Cellular community - prokaryotes
Cellular community - eukaryotes
Cell motility
Cell growth and death
Signaling molecules and interaction
Signal transduction
Membrane transport
Translation
Transcription
Replication and repair
Folding, sorting and degradation
Substance dependence
Neurodegenerative disease
Infectious disease: viral
Infectious disease: parasitic
Infectious disease: bacterial
Immune disease
Endocrine and metabolic disease
Drug resistance: antineoplastic
Drug resistance: antimicrobial
Cardiovascular disease
Cancer: specific types
Cancer: overview
Xenobiotics biodegradation and metabolism
Nucleotide metabolism
Metabolism of terpenoids and polyketides
Metabolism of other amino acids
Metabolism of cofactors and vitamins
Lipid metabolism
Glycan biosynthesis and metabolism
Energy metabolism
Carbohydrate metabolism
Biosynthesis of other secondary metabolites
Amino acid metabolism
Sensory system
Nervous system
Immune system
Excretory system
Environmental adaptation
Endocrine system
Digestive system
Development and regeneration
Circulatory system
Aging
Cellular Processes
Environmental Information Processing
Genetic Information Processing
Human Diseases
Metabolism
Organismal Systems
0 50 100 150 200 250 300 350 400 450 500 550
B
GXUF-2
Transport and catabolism
Cellular community - prokaryotes
Cellular community - eukaryotes
Cell motility
Cell growth and death
Signaling molecules and interaction
Signal transduction
Membrane transport
Translation
Transcription
Replication and repair
Folding, sorting and degradation
Substance dependence
Neurodegenerative disease
Infectious disease: viral
Infectious disease: parasitic
Infectious disease: bacterial
Immune disease
Endocrine and metabolic disease
Drug resistance: antineoplastic
Drug resistance: antimicrobial
Cardiovascular disease
Cancer: specific types
Cancer: overview
Xenobiotics biodegradation and metabolism
Nucleotide metabolism
Metabolism of terpenoids and polyketides
Metabolism of other amino acids
Metabolism of cofactors and vitamins
Lipid metabolism
Glycan biosynthesis and metabolism
Energy metabolism
Carbohydrate metabolism
Biosynthesis of other secondary metabolites
Amino acid metabolism
Sensory system
Nervous system
Immune system
Excretory system
Environmental adaptation
Endocrine system
Digestive system
Development and regeneration
Circulatory system
Aging
Cellular Processes
Environmental Information Processing
Genetic Information Processing
Human Diseases
Metabolism
Organismal Systems
0 50 100 150 200 250 300 350 400 450 500 550 600 650
C
GXUF-3
Transport and catabolism
Cellular community - prokaryotes
Cellular community - eukaryotes
Cell motility
Cell growth and death
Signaling molecules and interaction
Signal transduction
Membrane transport
Translation
Transcription
Replication and repair
Folding, sorting and degradation
Substance dependence
Neurodegenerative disease
Infectious disease: viral
Infectious disease: parasitic
Infectious disease: bacterial
Immune disease
Endocrine and metabolic disease
Drug resistance: antineoplastic
Drug resistance: antimicrobial
Cardiovascular disease
Cancer: specific types
Cancer: overview
Xenobiotics biodegradation and metabolism
Nucleotide metabolism
Metabolism of terpenoids and polyketides
Metabolism of other amino acids
Metabolism of cofactors and vitamins
Lipid metabolism
Glycan biosynthesis and metabolism
Energy metabolism
Carbohydrate metabolism
Biosynthesis of other secondary metabolites
Amino acid metabolism
Sensory system
Nervous system
Immune system
Excretory system
Environmental adaptation
Endocrine system
Digestive system
Development and regeneration
Circulatory system
Aging
Cellular Processes
Environmental Information Processing
Genetic Information Processing
Human Diseases
Metabolism
Organismal Systems
0 50 100 150 200 250 300 350 400 450 500 550 600
D
GXUF-1
181
58
64
15
28
54
0
3
51
187
1229
467
79
GXUF-2
197
65
84
16
34
52
0
3
59
197
1323
487
80
GXUF-3
186
61
77
16
36
52
0
3
58
192
1280
464
74
Cellular Processes
Environmental Information Processing
Genetic Information Processing
Human Diseases
Metabolism
Organismal Systems

Fig. S5 The KEGG metabolic pathway classification diagram.
A-C diagrams of GXUF-1, GXUF-2, and GXUF-3. **D** The respective KEGG primary classifications of three pathogens causing sugarcane root rot were analyzed for determining their shared genes.

Tryptophan metabolism

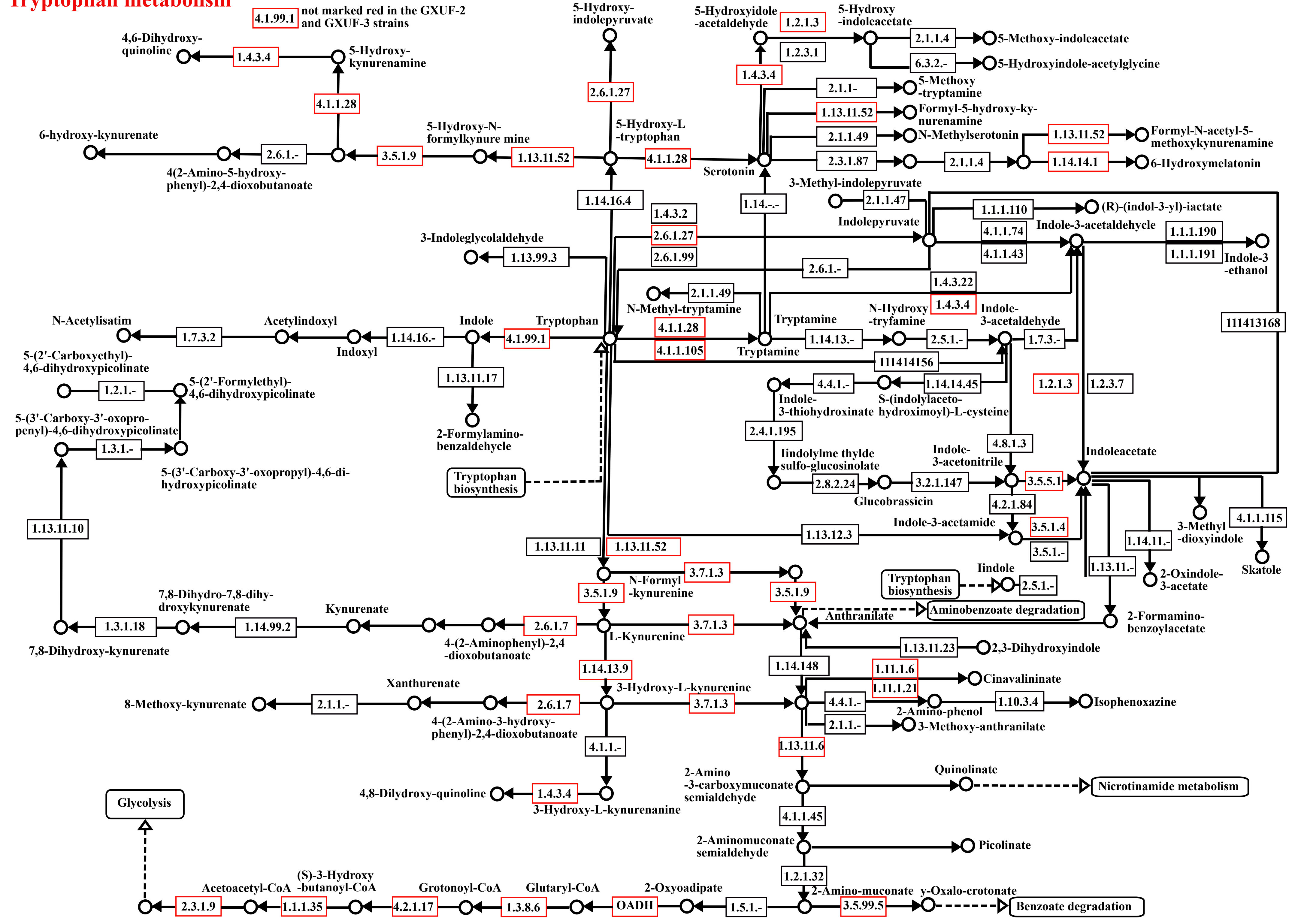

Fig. S6 Genes detected in KEGG map of tryptophan metabolism (00380).
According to the KEGG global metabolic network, the tryptophan metabolic pathway of these three pathogens causing sugarcane root rot was analyzed. Red borders represent disease associated genetic variants.

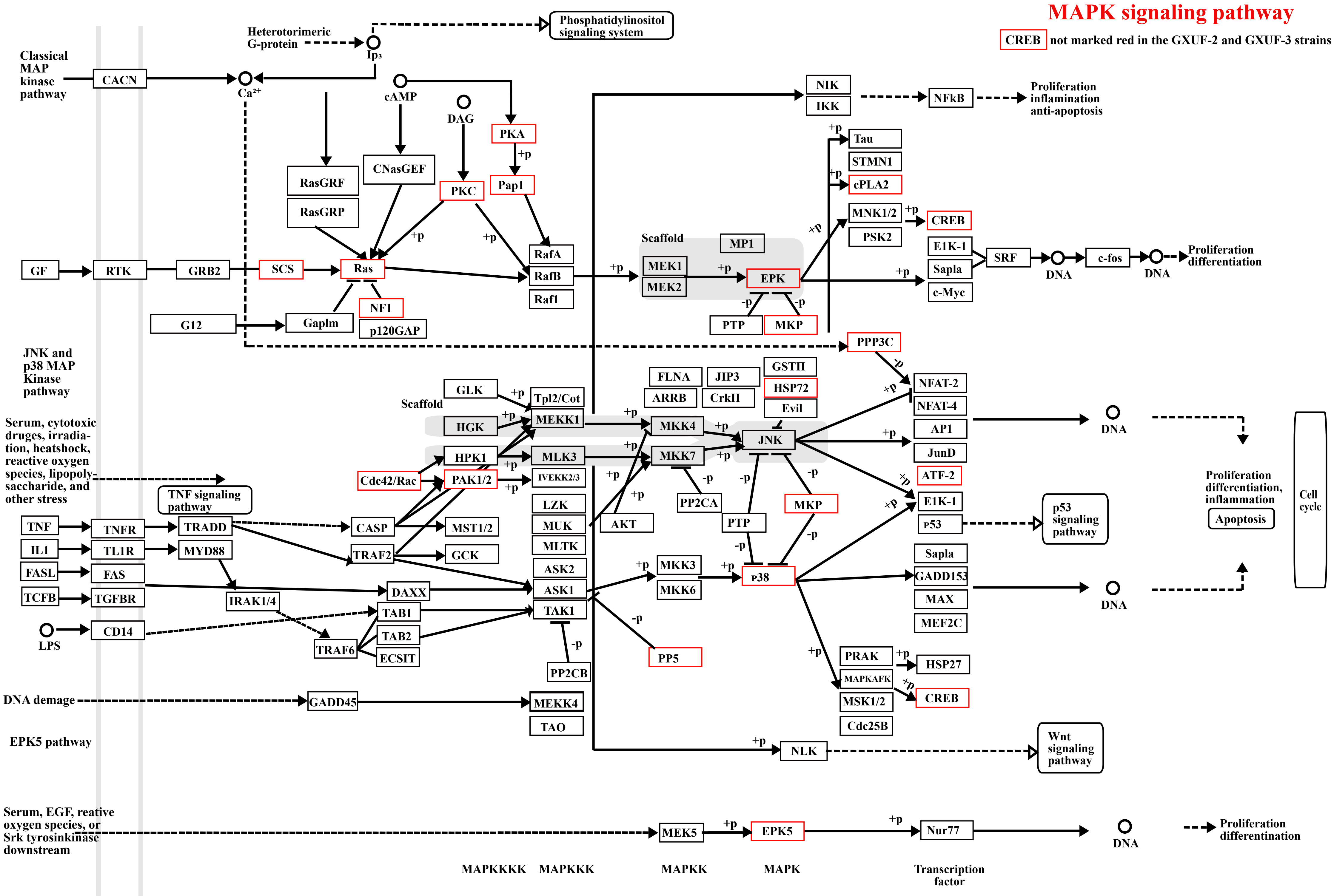
MAPK signaling pathway
CREB not marked red in the GXUF-2 and GXUF-3 strains
Classical MAP kinase pathway
CACN
Ca2+
Heterotorimeric G-protein
Ip3
Phosphatidylinositol signaling system
cAMP
DAG
PKA
Pap1
PKC
RasGRF
RasGRP
CNasGEF
GF
RTK
GRB2
SCS
Ras
NF1
p120GAP
Gaplm
G12
RafA
RafB
Raf1
Scaffold
MP1
MEK1
MEK2
EPK
PTP
MKP
NIK
IKK
NFkB
Proliferation inflamination anti-apoptosis
Tau
STMN1
cPLA2
MNK1/2
CREB
PSK2
E1K-1
Sapla
c-Myc
SRF
DNA
c-fos
Proliferation differentiation
PPP3C
JNK and p38 MAP Kinase pathway
Serum, cytotoxic druges, irradia-tion, heatshock, reactive oxygen species, lipopoly-saccharide, and other stress
GLK
Tpl2/Cot
HGK
MEKK1
HPK1
MLK3
Cdc42/Rac
PAK1/2
IVEKK2/3
LZK
MUK
MLTK
ASK2
ASK1
TAK1
FLNA
JIP3
ARRB
CrkII
GSTII
HSP72
Evil
MKK4
MKK7
JNK
PP2CA
AKT
PTP
MKP
NFAT-2
NFAT-4
AP1
JunD
ATF-2
E1K-1
P53
p53 signaling pathway
Proliferation differentiation, inflammation
Apoptosis
Cell cycle
TNF signaling pathway
TNF
TNFR
TRADD
IL1
TL1R
MYD88
FASL
FAS
TCFB
TGFBR
LPS
CD14
CASP
MST1/2
TRAF2
GCK
DAXX
IRAK1/4
TAB1
TAB2
TRAF6
ECSIT
PP2CB
MKK3
MKK6
p38
PP5
Sapla
GADD153
MAX
MEF2C
PRAK
HSP27
MAPKAFK
CREB
MSK1/2
Cdc25B
DNA demage
GADD45
MEKK4
TAO
EPK5 pathway
NLK
Wnt signaling pathway
Serum, EGF, reative oxygen species, or Srk tyrosinkinase downstream
MEK5
EPK5
Nur77
+p
-p
MAPKKKK
MAPKKK
MAPKK
MAPK
Transcription factor

Fig. S7 Genes detected in KEGG map of MAPK signaling pathway (04010).
According to the KEGG global metabolic network, the MAPK metabolic pathway of these three pathogens causing sugarcane root rot was analyzed. Red borders represent disease associated genetic variants.

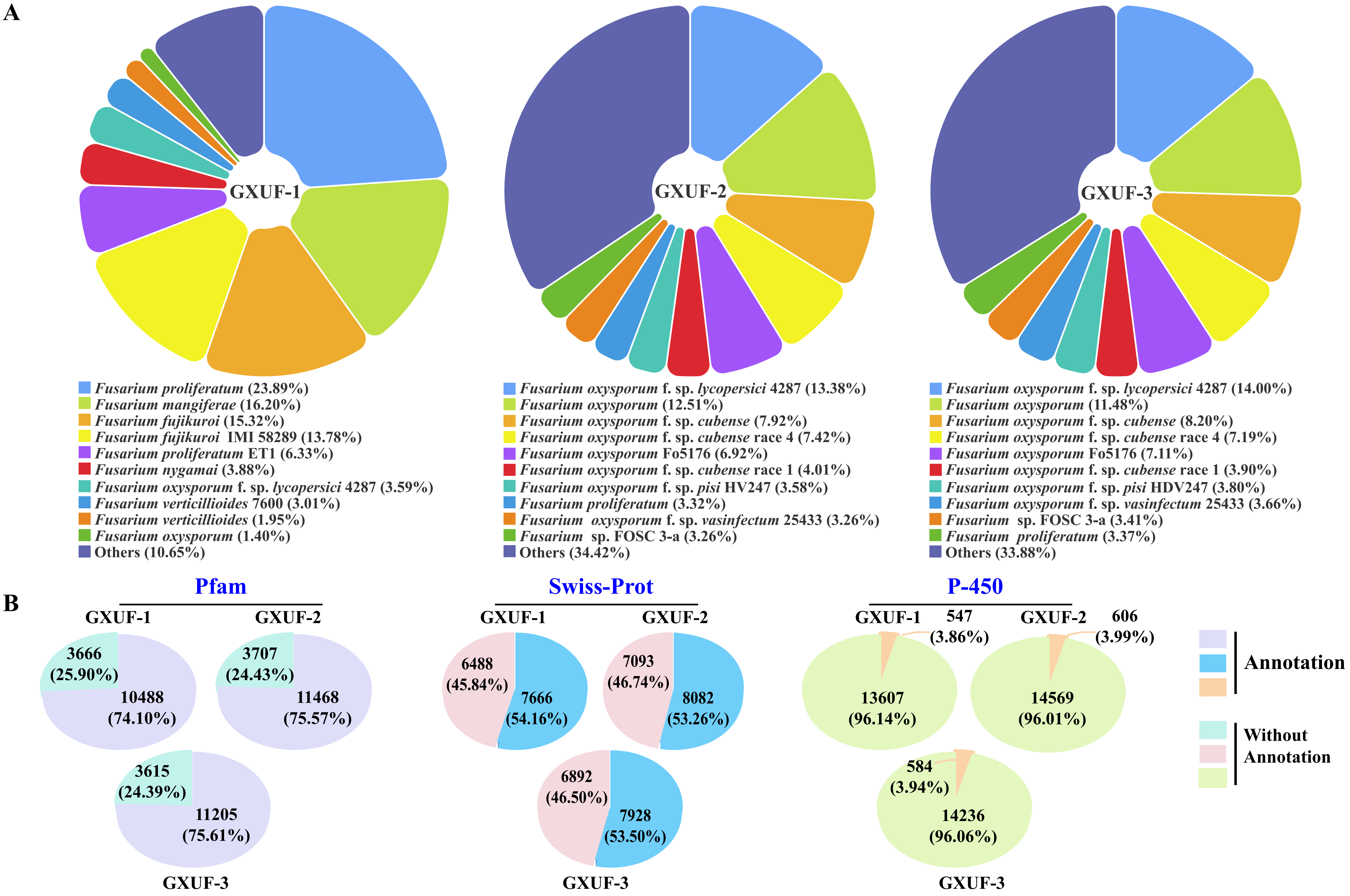

A
GXUF-1
GXUF-2
GXUF-3
Fusarium proliferatum (23.89%)
Fusarium mangiferae (16.20%)
Fusarium fujikuroi (15.32%)
Fusarium fujikuroi IMI 58289 (13.78%)
Fusarium proliferatum ET1 (6.33%)
Fusarium nygamai (3.88%)
Fusarium oxysporum f. sp. lycopersici 4287 (3.59%)
Fusarium verticillioides 7600 (3.01%)
Fusarium verticillioides (1.95%)
Fusarium oxysporum (1.40%)
Others (10.65%)
Fusarium oxysporum f. sp. lycopersici 4287 (13.38%)
Fusarium oxysporum (12.51%)
Fusarium oxysporum f. sp. cubense (7.92%)
Fusarium oxysporum f. sp. cubense race 4 (7.42%)
Fusarium oxysporum Fo5176 (6.92%)
Fusarium oxysporum f. sp. cubense race 1 (4.01%)
Fusarium oxysporum f. sp. pisi HV247 (3.58%)
Fusarium proliferatum (3.32%)
Fusarium oxysporum f. sp. vasinfectum 25433 (3.26%)
Fusarium sp. FOSC 3-a (3.26%)
Others (34.42%)
Fusarium oxysporum f. sp. lycopersici 4287 (14.00%)
Fusarium oxysporum (11.48%)
Fusarium oxysporum f. sp. cubense (8.20%)
Fusarium oxysporum f. sp. cubense race 4 (7.19%)
Fusarium oxysporum Fo5176 (7.11%)
Fusarium oxysporum f. sp. cubense race 1 (3.90%)
Fusarium oxysporum f. sp. pisi HDV247 (3.80%)
Fusarium oxysporum f. sp. vasinfectum 25433 (3.66%)
Fusarium sp. FOSC 3-a (3.41%)
Fusarium proliferatum (3.37%)
Others (33.88%)
B
Pfam
GXUF-1
GXUF-2
3666 (25.90%)
10488 (74.10%)
3707 (24.43%)
11468 (75.57%)
3615 (24.39%)
11205 (75.61%)
GXUF-3
Swiss-Prot
GXUF-1
GXUF-2
6488 (45.84%)
7666 (54.16%)
7093 (46.74%)
8082 (53.26%)
6892 (46.50%)
7928 (53.50%)
GXUF-3
P-450
GXUF-1
GXUF-2
547 (3.86%)
606 (3.99%)
13607 (96.14%)
14569 (96.01%)
584 (3.94%)
14236 (96.06%)
GXUF-3
Annotation
Without Annotation

Fig. S8 Statistics of gene annotation using general databases.
A The NR database annotation statistics of GXUF-1 (left), GXUF-2 (middle), and GXUF-3 (right) causing sugarcane root rot. **B** The pie chart representing the number of annotated genes of three pathogens causing sugarcane root rot in the common databases, i.e., Protein families database of alignments and hidden Markov models (Pfam) (left), Swiss-Prot (middle), and Cytochrome P-450 (P-450) (right).
